# *Helicobacter pylori* type 4 secretion systems as gastroduodenal disease markers

**DOI:** 10.1038/s41598-021-83862-1

**Published:** 2021-02-25

**Authors:** Bui Hoang Phuc, Vo Phuoc Tuan, Ho Dang Quy Dung, Tran Thanh Binh, Pham Huu Tung, Tran Dinh Tri, Ngo Phuong Minh Thuan, Vu Van Khien, Tran Thi Huyen Trang, Junko Akada, Takeshi Matsumoto, Yoshio Yamaoka

**Affiliations:** 1 Department of Environmental and Preventive Medicine, Faculty of Medicine, Oita University, Yufu, 879-5593 Japan; 2 Department of Microbiology, Cho Ray Hospital, Ho Chi Minh City, 749000 Vietnam; 3 Department of Endoscopy, Cho Ray Hospital, Ho Chi Minh City, 749000 Vietnam; 4 Department of Hepatogastroenterology, 108 Military Central Hospital, Hanoi, 749000 Vietnam; 5 Department of Molecular Biology, 108 Military Central Hospital, Hanoi, 749000 Vietnam; 6 Department of Medicine, Gastroenterology and Hepatology Section, Baylor College of Medicine, Houston, 77030 TX USA; 7 Global Oita Medical Advanced Research Center for Health, Oita University, Yufu, 879-5593 Japan

**Keywords:** Microbiology, Medical research

## Abstract

Although the type 4 secretion system of the integrating and conjugative elements *(tfs* ICE) is common in *Helicobacter pylori*, its clinical association with the *cag* pathogenicity island (cagPAI) have not yet been well-investigated. In this study, Vietnamese patient *H. pylori* samples (46 duodenal ulcer (DU), 51 non-cardia gastric cancer (NCGC), 39 chronic gastritis (CG)) were fully sequenced using next-generation sequencing and assembled into contigs. *tfs3, tfs4,* and cagPAI genes were compared with the public database. Most (94%) *H. pylori* strains possessed a complete cagPAI, which was the greatest risk factor for clinical outcomes, while the prevalences of *tfs3* and *tfs4* were 45% and 77%, respectively. Complete *tfs3* and *tfs4* were found in 18.3% and 17.6% of strains, respectively. The prevalence of *H. pylori* strains with complete *tfs3* ICE in DU patients was significantly higher than that in NCGC patients (30.4% vs 11.7%, *P* < 0.05). In addition, the prevalence of strains with complete *tfs3* ICE and cagPAI was significantly higher in DU patients than that in NCGC (28.4% vs 9.8%, *P* = 0.038) and CG patients (28.2% vs 7.7%, *P* = 0.024). cagPAI and complete *tfs3* increased the risk of DU compared to NCGC (OR = 3.56, 95%CI: 1.1–14.1, *P* = 0.038) and CG (OR = 4.64, 95%CI: 1.1–27.6, *P* = 0.024). A complete cluster of *tfs3* ICE was associated with gastroduodenal diseases in Vietnam. However, there was a low prevalence of the *dupA*/complete *dupA* cluster (15.4%) in the Vietnam strains. The prevalence of cagPAI in Vietnam strains was significantly higher than in US (*P* = 0.01) and Indonesia (*P* < 0.0001); the prevalence of the *dupA* cluster was also higher in the Vietnam strains than in the Indonesian strains (*P* < 0.05). In addition, the prevalence of *ctkA*, an accessory gene of *tfs3*, was significantly different between Vietnam and US strains (28% vs 2%, *P* = 0.0002). In summary, the acquisition of *tfs3/4* ICE was common in *H. pylori* strains in patients with gastroduodenal disease in Vietnam, and the complete cluster of *tfs3* ICE was a reliable marker for the severity of disease in the *H. pylori* infected population.

## Introduction

*Helicobacter pylori* is a gram-negative bacterium that consistently infects the human stomach. The prevalence of *H. pylori* infection in the human population is approximately 50% and is responsible for a subset of 15–20% of clinical cases, with less than 5% of patients developing gastric cancer^1,2^. Together with the environment and host factors, the virulence of *H. pylori* is believed to be an important factor increasing the risk of gastroduodenal diseases^3^.

The type 4 secretion system (T4SS) is a protein complex found in prokaryotes used to transport DNA, proteins, or effector molecules from the cytoplasm to the extracellular space beyond the cell. The *H. pylori* genome is known to encode up to four T4SSs, and each plays an independent role during host infection^4^. The first discovered T4SS was located within the *cag* pathogenicity island (cagPAI), which plays a crucial role in *H. pylori* infection by forming a T4SS assembly, interacting with integrin receptor (α5β1), and supporting the injection of CagA, an oncogenic protein of *H. pylori*, into the host cell^5,6^. After translocating into gastric epithelial cells, CagA localized to the inner surface of the plasma membrane, in which it undergoes tyrosine phosphorylation at the Glu-Pro-Ile-Tyr-Ala (EPIYA) motif^7^. The length of the cagPAI is about 40 kb and is comprised of 26 to 30 genes, depending on the strains that encode a complete set of T4SS (*virB1-virB11*), a coupling-protein *virD4*, and a subset of the *cag* genes, which contribute to a functional cagPAI^8^. The presence of the cagPAI had been found to be associated with severe inflammation and the development of gastroduodenal diseases^6^. The second T4SS was named as *comB*, which comprises all T4SS core components (*comB2* to *comB4*, and *comB6* to *comB10*) and plays an important role during the natural transformation of *H. pylori*^9^. The DNA transformation and homologous recombination substantially maintain the genome variability, which is crucial for promoting chronic infection of *H. pylori*^10^. In a different manner than the other T4SSs, *comB*-T4SS was unique in DNA uptake, especially in the efficiency of DNA transfer, which enabled the survival of *H. pylori* during long-term infection^10^. The third T4SS was previously called TnPZ (transposon element of plasticity region), and had been identified within the plasticity regions in which the GC content was lower (34–35%) than that in the rest of the genome (39%)^11,12^. The full length of TnPZ was determined to be 37 kb to 46 kb and is composed of a cluster of T4SS genes, a tyrosine recombinase family gene (*xerT*), a long open reading frame (> 2800 codon) encoded to helicase and DNA *methylase* domain, and a subset of accessory genes with unknown function^12^. Since there was evidence for horizontal gene transfer of TnPZ in a conjugation manner, they were termed as integrating and conjugating elements (ICE) and referred to as ICE*Hptfs* or *tfs* ICE^4,12,13^. Similar to cagPAI, *tfs* ICE possessed all core genes of T4SS (*virB2*-*virB4, virB6-virB11,* and *virD4* coupling-protein) in addition to a *virD2* relaxase^12^. Because of the nucleotide diversity between *vir* genes of *tfs* ICEs in *H. pylori* strains, they were classified into 2 types: *tfs*3 and *tfs4*^13^*.* Sequence analysis showed that the nucleotide diversity of *tfs3* ICE genes from *virB2* to *virB11* was highly similar among *H. pylori* strains^13^. In contrast, *tfs4* ICEs are divided into 3 subtypes: *4a*, *4b*, and *4c* based on the sequence diversity of *virB2, virB3, virB4, virB6, virB7,* and *virB8* discriminating *4a* to *4b,* while the diversity of *virB11, virD2*, and *virD4* discriminates *4a/4b* to *4c*^13^. However, each *H. pylori* strain differently presented the combination of *tfs4* modules, which included the left (L1/L2), center (C1/C2), and right (R1/R2) modules^14^. In addition, a full set of T4SS genes was located within each module: L1/L2 (*xerT, virB6*), C1/C2 (*virD2, virD4, virB11, virB10, virB9*), and R1/R2 (*virB2, virB3, virB4*), and the *tfs4* subtypes were thus classified based on the following module combinations: *tfs4a* (L2C1R2), *tfs4b* (L1C1R1), *tfs4a/4b* (L2C1R1) and *tfs4c* (L2C2R2)^14,15^.

Component genes of *tfs3* and *tfs4* are associated with the pro-inflammatory activity in the gastric mucosa and increased risk of gastroduodenal diseases^16,17^. We previously showed that *dupA*, a *virB4* homolog located within the right module (R1) of *tfs4b* ICE, was considered to be a specific marker for duodenal ulcer (DU)^18^. Furthermore, the presence of *dupA* in combination with its neighbor T4SS genes forming an intact *dupA* cluster (C1R1) might be a more reliable marker for disease risk than incomplete *dupA* cluster or *dupA* alone^19^. The in vitro study showed that the expression of some *tfs4b* T4SS genes (*virB2, virB4, virB8,* and *virB10*) was more up-regulated in response to low pH and contact with the human gastric cell line, which supported the role of *tfs4b* in host colonization^20^. These studies suggested that *tfs3* and *tfs4b* might form an alternative T4SS for DNA or effector protein in a similar manner to cagPAI and were considered to be virulence factors of *H. pylori. tfs3* and *tfs4b* ICEs could distribute differently, suggesting that the risk of these clusters in gastroduodenal diseases should be considered within in each country and ethnicity^12–15^. Although the association of *tfs4* ICE with gastroduodenal diseases has been determined in some countries, the clinical association of *tfs3* ICE with gastroduodenal diseases has not yet been adequately investigated. Moreover, a comprehensive study about the prevalence and status of *tfs3* and *tfs4* in clinical outcomes has not yet been conducted. Hence, we conducted a study to examine the distribution and status of *tfs3* and *tfs4* in *H. pylori* strains isolated from Vietnamese patients with gastroduodenal diseases including DU, non-cardia gastric cancer (NCGC), and chronic gastritis (CG).

The prevalence of *H. pylori* infection in Vietnam was reported to be 65.6%, and the incidence of gastric cancer in Vietnam was classified as an intermediate risk in Asia, but the highest in Southeast Asia (age- standardized rate (ASR) of gastric cancer, 16.3/100,00 in both sex)^21–23^. Although several previous studies have investigated the virulence factors in Vietnam, these studied only examined some well-known virulence factors (*cagA*, *vacA,* and several cagPAI genes)^23–25^. It is still unknown why some infected subjects develop severe diseases like DU and gastric cancer. Therefore, we aimed to investigate the association of *tfs* ICE and cagPAI with clinical outcomes in Vietnam via whole-genome analyses using next-generation sequencing.

## Results

### Sequence comparison of *vir* T4SS genes of *tfs3, tfs4,* cagPAI, and comB

The genomes of 136 *H. pylori* strains were newly assembled into 23–129 contigs (Supplementary-Table S1), and then *tfs3* and *tfs4* were identified by the common 12 genes: *xerT*, *virB2, virB3, virB4, virB6, virB7, virB8, virB9, virB10, virB11, virD2*, and *virD4* using the ABRICATE pipeline. Similarly, the cagPAI and *comB* were identified by 10 (*virB2-B11, virD4*) and 6 (*virB2-virB4, virB7-virB10*) T4SS genes by the same approach, respectively.

**Figure 1:**
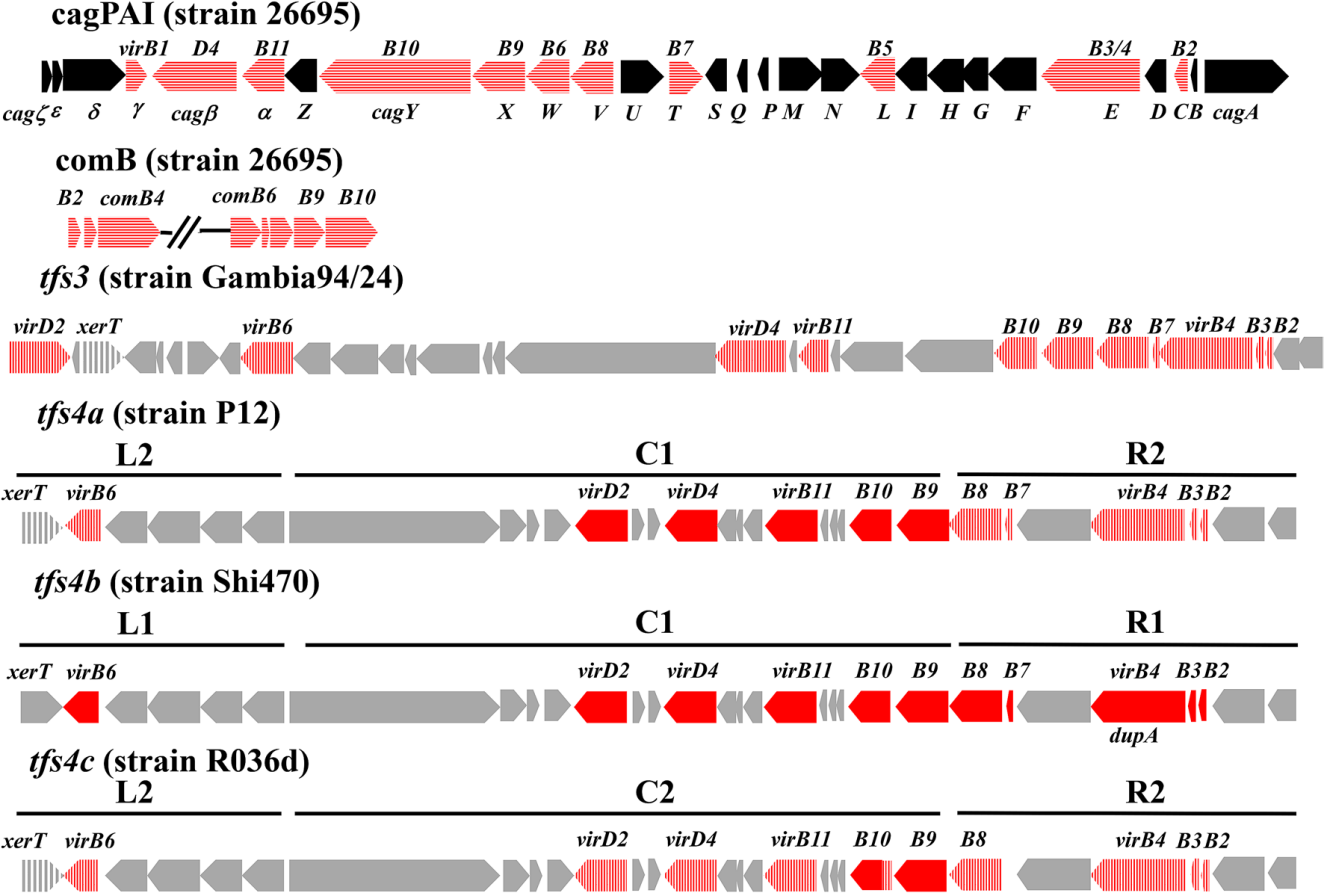
Gene arrangement of cagPAI, *tfs3*, *tfs4a*, *tfs4b*, and *tfs4c* ICE, which were retrieved from *H. pylori* strain 26,695, Gambia94/24, P12, Shi470, and R036d, respectively. The red color indicates *vir* homolog T4SS genes in which nucleotide identity is more than 80% to corresponding genes of *tfs4b* ICE*,* while horizontal dashed red and dashed gray colors indicate that nucleotide identity was less than 80%, respectively, in a sequence comparison between *tfs4b* and others (Table 1 and Table S4). The black color indicates the other *cag* genes of cagPAI (*ζ, ε, δ, Z, U, S, Q, P, M, N, I, H, G F, D, B, A*). Gray color indicates accessory genes of *tfs3* and *tfs4* ICE, which included DNA processing genes (*xerT* and *topA*) and unknown function genes.

Since all four types of *tfs* gene clusters contained *xerT* and 11 conserved *vir* genes, first, the nucleotide identities of each of these 12 genes of the *tfs3, tfs4a, tfs4b,* and *tfs4c* ICE were compared using pairwise sequence alignment. Their sequence identity is shown in Table S4 and visualized in Fig. 1. There was a low identity of T4SS nucleotide diversity between *tfs3* ICE and either of *tfs4a/b/c* ICE (less than 60%*)*. For *tfs3* ICE, there were only *virB6* and *virB7,* which was diverse in nucleotide sequence between strain Gambia94/24 and strain India7 (less than 80%), and the other *vir* T4SS genes had high identity (from 84–100%). We denoted *virB6* and *virB7* of strain Gambia94/24 as variant 1, while strain India7 possessed variant 2 of those. In contrast to *tfs3* ICE, there were only 2 T4SS genes, which were encoded by all three *tfs4* subtypes (*a/b/c)* with high identity: *virB9* with high identity (> 90%) and *virB10* with moderate identity (> 80%). The T4SS genes of *tfs4a* (strain P12) and *tfs4b* (strain Shi470) had high identity with more than 90% in *virB11, virD2*, and *virD4,* while *tfs4a* (strain P12) and *tfs4c* (strain R036d) shared identity between 83.5% and 98.0% in *virB2, virB3, virB4, virB6, virB8,* and *virB10*. Therefore, the nucleotide diversity in 3 genes (*virB11, virD2,* and *virD4*) could distinguish *tfs4a* and *tfs4b* from *tfs4c,* while that in 6 genes (*virB2, virB3, virB4, virB6, virB7, virB8*) could distinguish *tfs4a/4c* from *tfs4b.* From this analysis, we used *tfs3* (strains Gambia94/24 and India7), *tfs4a* (strain P12), *tfs4b* (strain Shi470), and *tfs4c* (strain R036d) to construct a reference database. These strains possessed the prototypical *tfs3/4a/b/c* in addition to cagPAI and comB clusters with no frameshift or premature stop codon in any gene. In addition, the module combinations of *tfs4* in these strains were L2C1R2, L1C1R1, L2C1R1, and L2C2R2 for *tfs4a*, *tfs4b*, *tfs4a/4b* and *tfs4c*, respectively^14,15^. The annotation number of these *tfs* genes was retrieved from reference strains from Genbank database and is shown in Table S2 (https://www.ncbi.nlm.nih.gov/nuccore).

Also, the 11 T4SS genes in *tfs3* ICE (strain Gambia94/24), *tfs4a* ICE (strain P12), *tfs4b* ICE (strain Shi470), and *tfs4c* (strain R036d) were applied for pairwise sequence comparison to double-check identity against *comB* and cagPAI, the other two T4SS clusters, in the same strain background. The low identity from the pairwise alignment of T4SS nucleotide sequences of *tfs3/tfs4a/tfs4b/tfs4c* against *comB* (less than 61%) and cagPAI (less than 50%), indicated the distinct evolution from *tfs3* and *tfs4* ICE (Table S3 – Supplementary).

### Prevalence *of tfs3*, *tfs4*, cagPAI, and comB in Vietnam isolates

Next, all 136 strains were applied to identify the T4SS genes of *tfs3*, *tfs4a/b/c*, cagPAI, and *comB* using the ABRICATE pipeline and the reference sequences from the above step. The sequence was identified and denoted as *tfs3, tfs4*, cagPAI, or *comB* based on whether the coverage was more than 60% and percentage identity more than 80% against the query. Based on the criteria from the above analysis, the subtypes of *tfs4 (a/b/c)* and *tfs4* modules (L1/L2/C1/C2/R1/R2) were also determined. As shown in Table S5, the number of strains possessing *tfs3* and *tfs4* was 62 (45.5%) and 105 (77%), respectively. These results indicated the high prevalence of *tfs3/4* in *H. pylori* strains isolated from gastroduodenal patients in Vietnam. The distribution of the *tfs4* module in Vietnam strains skewed towards L1 (30.1%) and L2 (47.0%) compared to R1 (19.8%) and R2 (27.9%); the C1 module (29.4%) was more prevalent than the C2 module (2.9%). The number and percentage identity of each *vir* T4SS gene of *tfs3*, *tfs4* are also shown in Table S5.

In this study, we selected all 11 *vir* genes to access the status of cagPAI. Based on the criteria, 128 (94.1%) strains possessed the complete cagPAI and 2 strains had incomplete cagPAI (1.4%), while only 6 strains were cagPAI–negative (4.4%). Also, all strains possessed the comB cluster in their genome. The coverage and identity percentage of each *tfs3, tfs4,* cagPAI, and comB T4SS gene in each strain are shown in the additional data (Table S6).

### Distribution of the *cagA*-EPIYA motif in terms of disease severity and geographical population

**Figure 2:**
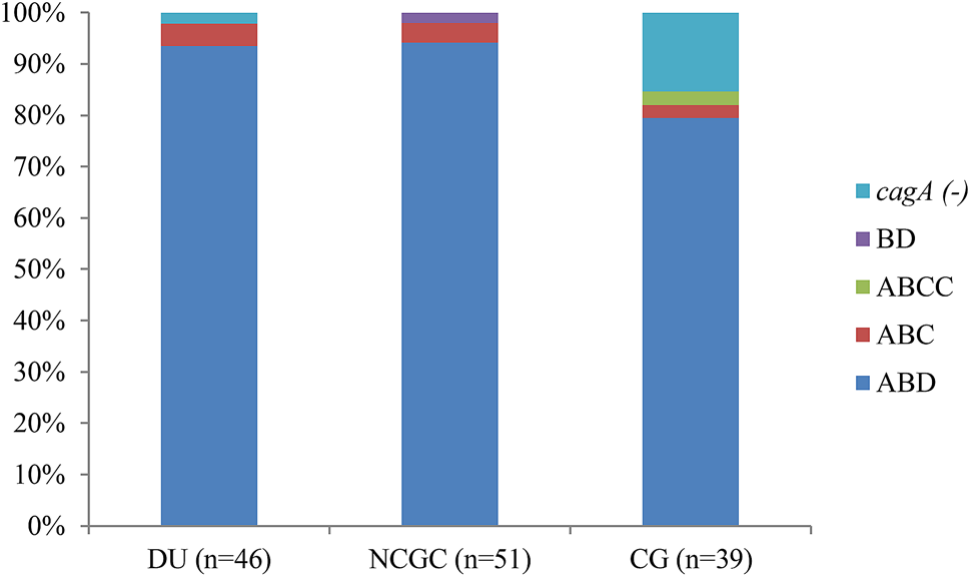
Distribution of *cagA*-EPIYA motifs in three clinical outcomes: duodenal ulcer (DU), non-cardia gastric cancer (NCGC), and chronic gastritis (CG). The EPIYA motifs of *cagA* included ABD, BD, ABC, and ABCC.

The *cagA* gene mostly harbored the ABD-type Glu-Pro-Ile-Tyr-Ala (EPIYA) motif: 43 (93%) in strains from DU patients, 48 (94%) in strains from NCGC patients, and 31 (79%) in strains from CG patients (Fig. 2). In contrast, a minority of patients harbored BD- (1 in NCGC), ABC- (2 DU, 2 NCGC, and 1 CG), and ABCC- (1 in CG) type EPIYA motifs. Seven strains were *cagA*-negative: 1 (2%) from DU patients and 6 (15.3%) from CG patients. The presence of *cagA*, regardless of the EPIYA motif, was associated with the severity of clinical outcomes in Vietnam: 97% in DU vs 84% in CG (*P* = 0.04) and 100% in NCGC vs 84% in CG (*P* = 0.005).

**Figure 3:**
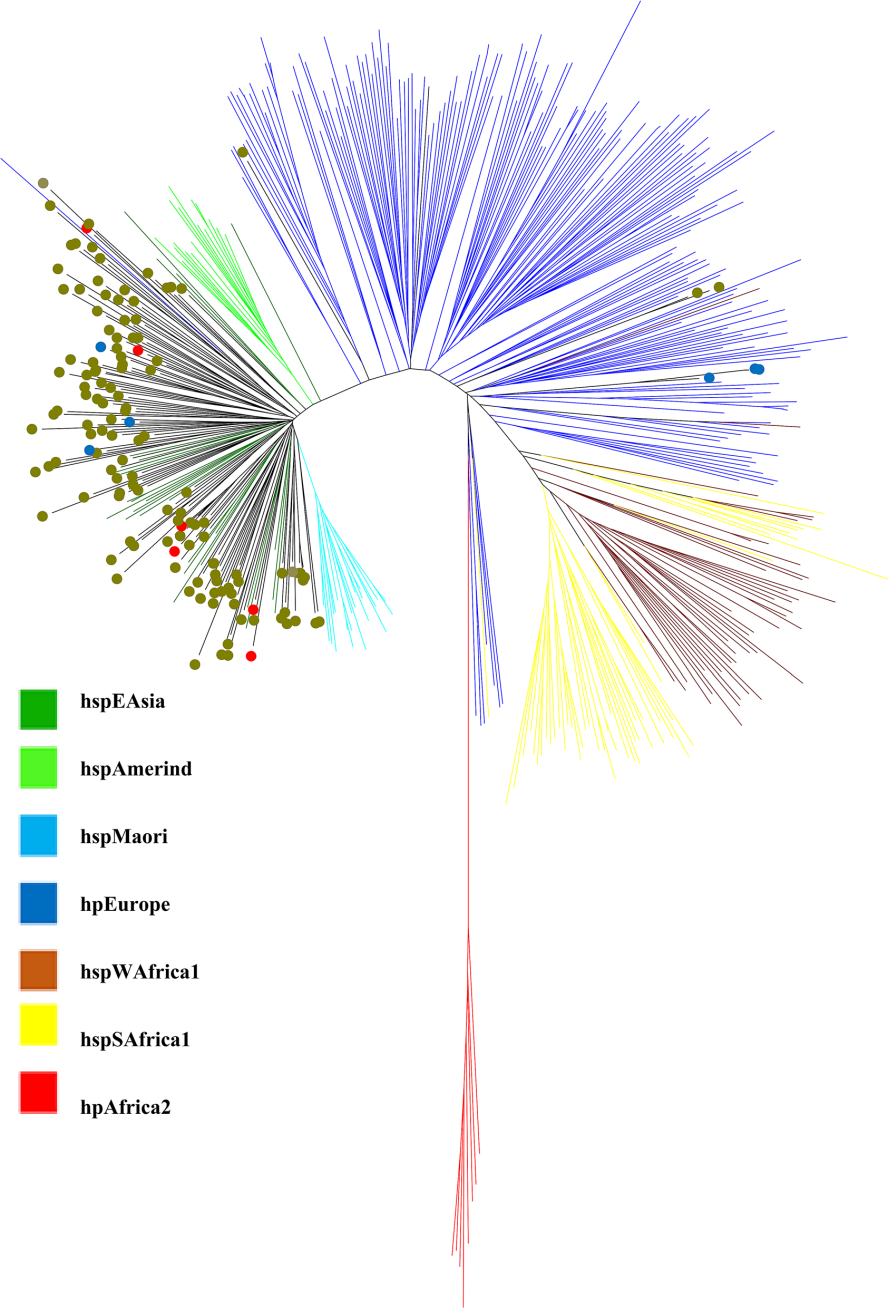
A phylogenetic tree was constructed through the concatenation of seven housekeeping genes from 136 Vietnam strains (olive circle) and 379 reference strains from PubMLST (http://pubmlst.org/helicobacter/). The circle indicates our studied strains: blue (*cagA*-negative), red (Western-type cagA), and olive (East Asian-type *cagA*).

The distribution of the *cagA*-EPIYA motifs was mostly in agreement with the population-specificity of Vietnam strains: 124 (95.3%) of the hspEAsia strains harbored the East Asian-type *cagA*, and three (50%) of the hpEurope strains were cagPAI (-)/*cagA*(-) (Fig. 3). However, there was an exchange in the population-specific *cagA* genotype: 3 of 6 hpEurope strains possessed East Asian-type *cagA*, while 6 of 130 hspEAsia strains possessed Western-type *cagA* (Fig. 3).

### Distribution and status of *tfs3* and *tfs4a/b/c* in *H. pylori* isolated from patients with gastroduodenal diseases in Vietnam

As for the cagPAI, the strains that possessed all 11 *vir* T4SS genes (*virB2, virB3, virB4, virB6, virB7, virB8, virB9, virB10, virB11, virD2,* and *virD4*) were defined as a complete form (Table S5), while the other cases were an incomplete form. In contrast, the strain was defined as *tfs*-negative if *tfs* sequences were absent.

Based on this criterion, there was a high prevalence of *tfs*-positive strains in Vietnam (90.4%): DU (87.0%), NCGC (98.0%), and CG (84.6%) (Table 1). There was a dominant prevalent of single *tfs* compared to double *tfs* (58.1% vs 32.3%, *P* < 0.001), which were divided into: *tfs3* (13.2%), *tfs4* (44.8%), and *tfs3-4* (32.3%). Together with the high prevalence of *tfs3* and *tfs4*, the co-existence of *tfs3-4* would be a result of horizontal gene transfer during *H. pylori* mixed-infection in the host. The prevalence of *tfs3* was similar between DU (50%), CG (41.1%), and NCGC (46.1%) patients, while the prevalence of *tfs4* was higher in NCGC (88.2%) and CG (74.3%) patients than in DU (67.4%) patients. Considering the *tfs4* module, there were no differences in the prevalences of the L2/C2/R2/L1/C1 module between DU, NCGC, and CG patients. In contrast, the prevalence of R1 module was significantly different between the NCGC and CG patients and the DU patients (25.5% vs 8.7% and 25.6% vs 8.7%, respectively, all *P* < 0.05).

**Table 1:** Distribution of *tfs3* and *tfs4* ICE in DU, NCGC, and CG patients.

Type of T4SS	DU (%), n = 46		NCGC (%), n = 51		CG (%), n = 39		Total (%), n = 136	
*tfs* negative	6 (13.0)		1 (0.2)		6 (15.3)		13 (9.6)	
*tfs* positive	40 (87.0)		50 (98.0)		33 (84.6)		123 (90.4)	
Incomplete *tfs* | Complete tfs*	21 (45.6)	19 (41.3)	35 (68.6)	15 (29.4)	19 (48.7)	14 (35.9)	75 (55.1)	48 (35.3)
Single *tfs*								
*tfs3*	2 (4.3)	7 (15.2)	2 (3.9)	3 (5.8)	4 (10.2)	0	8 (5.8)	10 (7.3)
*tfs4*	13 (28.2)	4 (8.7)	20 (39.2)	9 (17.6)	8 (20.5)	7 (17.9)	41 (30.1)	20 (14.7)
Double tfs**								
Incomplete *tfs3* and *tfs4 |* Complete *tfs3/tfs4*	6 (13.0)	8 (17.4)	13 (25.5)	3 (5.8)	7 (17.9)	7 (17.9)	26 (19.1)	18 (13.2)
*tfs4* module								
L1	10 (21.0)		18 (35.3)		13 (33.3)		41 (30.1)	
L2	21 (45.6)		27 (52.9)		16 (41.0)		64 (47.0)	
C1	9 (19.5)		16 (31.3)		15 (38.4)		40 (29.4)	
C2	2 (4.3)		0		2 (5.1)		4 (2.9)	
R1	4 (8.7)		13 (25.5)^a^		10 (25.6)^b^		27 (19.8)	
R2	14 (30.4)		14 (27.4)		10 (25.6)		38 (27.9)	
***Other T4SS***								
*cag*PAI	45 (97.8)		51 (100)		34 (87.1)		130 (96.1)	
comB***	46 (100)		51 (100)		39 (100)		136 (100)	

80% < Identity of gene only counted.

Complete tfs harbored 11 vir genes (virB2, virB3, virB4, virB6, virB7, virB8, virB9, virB10, virB11, virD4, and virD2)

** Double *tfs* were divided into: both of complete *tfs,* one of complete *tfs,* and both of *tfs* incomplete form cagPAI harbored all ten *vir* genes (*virB2, virB3, virB4, virB6, virB7, virB8, virB9, virB10, virB11,* and *virD4*).

***comB harbored all seven *vir* genes (*virB2, virB3, virB4, virB7, virB8, virB9,* and *virB10*).

^a^Indicated the significant different between NCGC to DU at *P* < 0.05.

^b^Indicated the significant different between CG to DU at *P* < 0.05.

Furthermore, we assessed the status of *tfs* among different types. Among 136 strains, the complete forms of *tfs3* and *tfs4* were observed in 18% and 19.1% of strains. The prevalences of the complete *tfs4* forms were as follows: 15.4% for L1C1R1, 0.7% for L2C1R1, and 2.9% for L2C2R2.

### The prevalence of *vir* T4SS genes of the *tfs3* cluster in gastroduodenal diseases

Although more than 45% of Vietnam strains possessed *tfs3* ICE, they dominantly harbored the incomplete form or only a fragment in the genome (Table 2). Among these incomplete *tfs3* clusters, there was a tendency to have the *xerT, virD2,* and *virB6,* while the *virB2-virD4* gene cluster was lost. Moreover, it is possible that the complete form of *tfs3* ICE formed an alternative functional assembly compared to the incomplete form or fragment. The prevalence of complete *tfs3* was significantly higher in DU patients compared to NCGC patients (30.4% vs 11.7%, *P* = 0.026) and CG patients (30.4% vs 12.8%, *P* = 0.068). This result showed the association of *tfs3* with gastroduodenal diseases in Vietnam. In addition, we assessed the status of both *tfs3* ICE and cagPAI. Interestingly, the prevalences of strains that possessed both cagPAI and complete *tfs3* was significant higher in DU patients compared to that in CG (28.2% vs 7.7%, *P* = 0.024) and NCGC (28.2% vs 9.8%, *P* = 0.038) patients.

**Table 2:** Prevalence of *tfs3* ICE T4SS genes in DU, NCGC, and CG.

	DU (%), n = 46	NCGC (%), n = 51	CG (%), n = 39	Total (%), n = 136
*virB2*	15 (32.6)	11 (21.5)	10 (25.6)	36 (26.4)
*virB3*	15 (32.6)	11 (21.5)	10 (25.6)	36 (26.4)
*virB4*	15 (32.6)	11 (21.5)	10 (25.6)	36 (26.4)
*virB6*	18 (39.1)	14 (27.4)	13 (33.3)	45 (33.0)
*virB7*	16 (34.7)	9 (17.6)	7 (17.9)	32 (23.5)
*virB8*	15 (32.6)	9 (17.6)	7 (17.9)	31 (22.8)
*virB9*	17 (36.9)	10 (19.6)	7 (17.9)	34 (25.0)
*virB10*	17 (36.9)	10 (19.6)	7 (17.9)	34 (25.0)
*virB11*	16 (34.8)	11 (21.5)	7 (17.9)	34 (25.0)
*virD4*	16 (34.8)	11 (21.5)	7 (17.9)	34 (25.0)
*virD2*	19 (41.3)	10 (19.6)	15 (38.4)	44 (32.3)
*xerT*	21 (45.6)	20 (39.2)	18 (46.1)	59 (43.3)
*ctkA*	14 (30.4)	15 (29.4)	10 (25.6)	39 (28.6)
Incomplete *tfs3*	9 (19.5)	15 (30.0)	13 (33.3)	37 (27.2)
Complete *tfs3*	14^b^ (30.4)	6 (11.7)	5 (12.8)	25 (18.4)
Complete *tfs3* and *ctkA*	7 (15.2)	5 (9.8)	1 (2.5)	12 (8.8)
Incomplete *tfs3* and cagPAI	9 (19.5)	13 (25.5)	11 (28.2)	33 (24.3)
Complete *tfs3* and cagPAI	13^a,b^ (28.2)	5 (9.8)	3 (7.7)	21 (15.4)

^a^Indicated significant difference in prevalence between DU and CG at *P* < 0.05.

^b^Indicated significant difference in prevalence between DU and NCGC at *P* < 0.05.

In addition, the prevalence of *ctkA* (cell-translocating kinase A)*,* an accessory gene of *tfs3* ICE, was 28.6% in Vietnam strains; this prevalence was divided into 23.9% for DU patients, 29.4% for NCGC patients, and 25.6% for CG patients. According to Delahay et al., the pro-inflammatory activity of *ctkA* was supported by the T4SS genes of *tfs3* ICE^14^. In our study, the prevalence of *ctkA*( +)/complete *tfs3* ICE tended to be higher in DU patients than in CG patients (15.2% vs 2.5%, *P* = 0.064) but not higher in NCGC patients than in CG patients (9.8% vs 2.5%, *P* = 0.228).

### Prevalence of *dupA* and its neighboring *vir* homologous genes in the *tfs4b* cluster in gastroduodenal diseases

The *dupA* cluster, which included the C1 and R1 modules, was combined with the L1 module forming *tfs4b* (L1C1R1) in 23 strains (16.9%) or the L2 module forming L2C1R1 in 4 strains (2.9%) in our study (Table 3). In contrast to *tfs3*, the complete *dupA* cluster (15.4%) was more prevalent than the incomplete form (4.4%) (Table 3). The incomplete *dupA* cluster tended to have the L1/L2 module (*xerT* and *virB6*) and, to a lesser extent, the C1 module (*virD2, virD4, virB11*, *virB10*, and *virB9*)*,* while the R1 module (*virB2, virB3,* and *virB4*) was almost absent. It has been confirmed that *dupA* is the *virB4* of *tfs4b* ICE, and the presence of *dupA* and its neighboring *vir* homologous genes (from *virB2* to *virD2*), which form a complete *dupA* cluster, is a more reliable disease marker than the presence of *dupA* alone^19^. Considering each disease group, a complete *dupA* cluster was found in 15.4% of the total strains (8.7% in DU, 17.6% in NCGC, and 20.5% in CG). In addition, the prevalence of strains with complete *dupA* cluster and cagPAI was 14.7% in Vietnamese patients, 8.7% in DU patients, 17.6% in NCGC patients, and 17.9% in CG patients.

**Table 3:** Prevalence of *dupA* cluster in DU, NCGC, and CG.

Module	T4SS genes	DU (%), n = 46	NCGC (%), n = 51	CG (%), n = 39	Total (%), n = 136
L1 (n = 23)	*xerT*	4 (8.7)	9 (17.6)	10 (25.6)	23 (16.9)
	*virB6*	4 (8.7)	9 (17.6)	10 (25.6)	23 (16.9)
L2 (n = 4)	*xerT*	0	3 (5.8)	1 (2.5)	4 (2.9)
	*virB6*	0	3 (5.8)	1 (2.5)	4 (2.9)
C1 (n = 27)	*virD2*	4 (8.7)	13 (25.5)	10 (25.6)	27 (19.8)
	*virD4*	4 (8.7)	10 (25.6)	9 (23.0)	23 (16.9)
	*virB11*	4 (8.7)	10 (25.6)	9 (23.0)	23 (16.9)
	*virB10*	4 (8.7)	11 (21.5)	10 (25.6)	25 (18.3)
	*virB9*	4 (8.7)	12 (23.5)	10 (25.6)	26 (19.1)
R1 (n = 27)	*virB8*	4 (8.7)	12 (23.5)	10 (25.6)	26 (19.1)
	*virB7*	4 (8.7)	13 (25.5)	10 (25.6)	27 (19.8)
	*virB4 (dupA)*	4 (8.7)	9 (17.6)	8 (20.5)	21 (15.4)
	*virB3*	4 (8.7)	9 (17.6)	8 (20.5)	21 (15.4)
	*virB2*	4 (8.7)	9 (17.6)	8 (20.5)	21 (15.4)
Incomplete *dupA* cluster		0	4 (7.8)	2 (5.1)	6 (4.4)
Complete *dupA* cluster		4 (8.7)	9 (17.6)	8 (20.5)	21 (15.4)
Incomplete dupA cluster and cagPAI		0	3 (5.8)	1 (2.5)	4 (2.9)
Complete dupA cluster and cagPAI		4 (8.7)	9 (17.6)	7 (17.9)	20 (14.7)

Previous studies showed that the long-intact form of *dupA* (2499 bp) with no mutation, which caused a frameshift or premature stop codon in the sequence, was determined to be associated with the severity of clinical outcomes in comparison to the short-form *dupA*^26^*.* Among 21 *dupA*-positive strains (15.4%), there were 10 strains (7.3%) possessing the long intact-*dupA,* while 11 strains (8.1%) possessed the mutation, which might be the non-functional *dupA.* The type of *dupA* mutation and gastroduodenal diseases are shown in Table 4. Non-intact *dupA* was more frequently observed in NCGC (4/51; 7.8%) and CG patients (6/39; 15.3%) but not in DU patients (1/46; 2.2%). In contrast, the prevalence of long-intact *dupA* was divided into: DU (3/46; 6.5%), NCGC (5/51; 9.8%), and CG (2/39; 5.1%).

**Table 4:** The mutation of *dupA* in gastroduodenal patients in Vietnam.

Strains	Mutation	Type of mutation	Diseases
VN0355	E750*	Premature stop codon	NCGC
VN0434	E65*	Premature stop codon	NCGC
VN0448	S291fs	Frameshift	NCGC
VN0472	D601fs	Frameshift	NCGC
VN0754	2030_2256del	Deletion	DU
VN1158	F397fs	Frameshift	CG
VN1165	G458fs	Frameshift	CG
VN1192	G458fs	Frameshift	CG
VN1196	G434fs	Frameshift	CG
VN1212	K113fs	Frameshift	CG
VN1251	494_528del	Deletion	CG

### Comparison of *tfs3, dupA* cluster, and cagPAI prevalence between Vietnam and the other geographical regions

**Figure 4:**
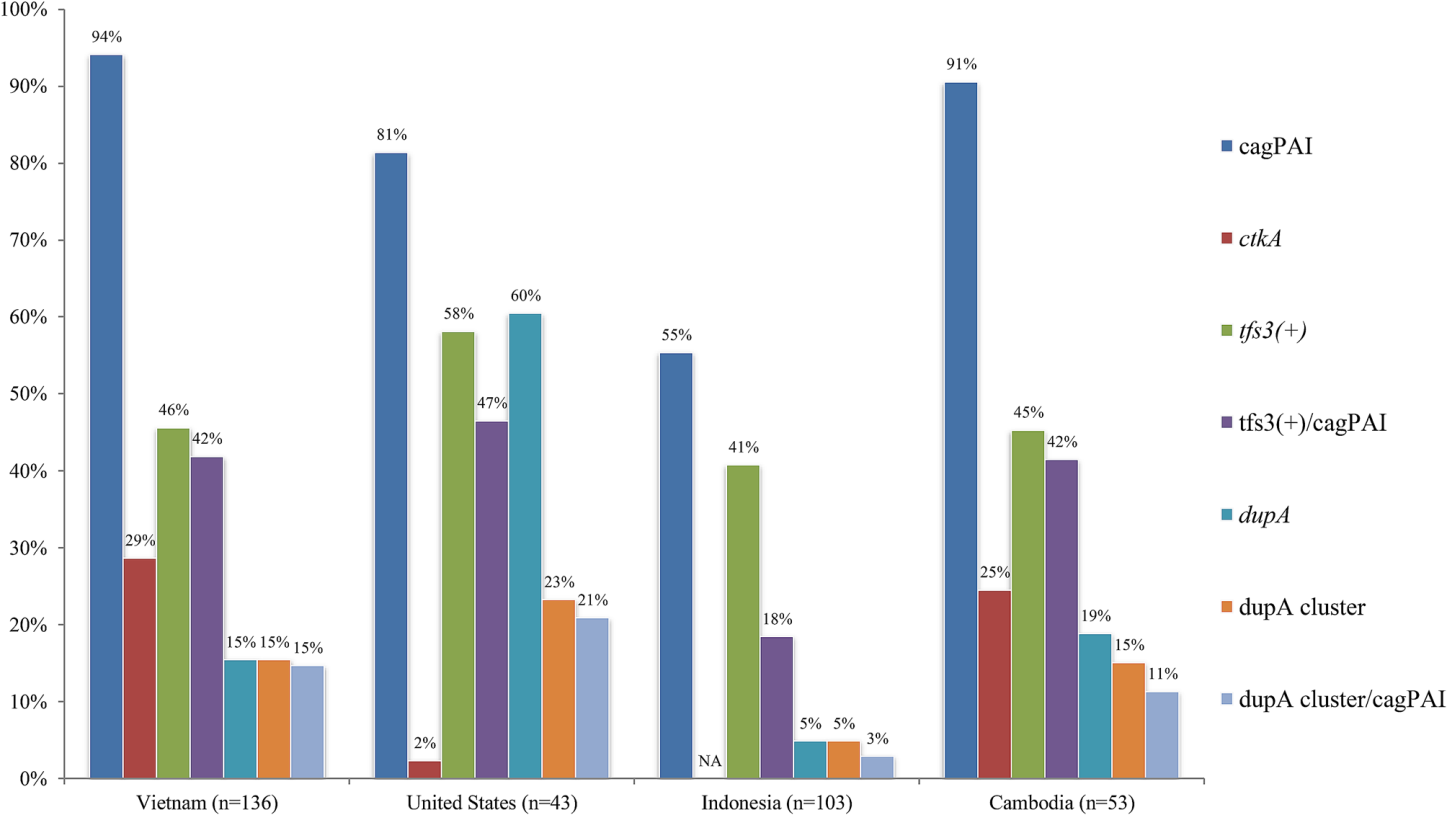
Distribution of *tfs3*, *dupA* cluster, and cagPAI across four geographical regions: Vietnam, Indonesia, Cambodia, and the US (Bronx, NY). The prevalences of *ctkA* and *dupA* were included for the comparison.

The prevalence of cagPAI, *tfs3,* and *dupA* cluster in Vietnam strains was compared to those in strains from other geographical regions: Indonesia^15^, Cambodia (KH)^27^, and US (MPH)^28^ strains (Table S6). There were no cases of gastric cancer in previous studies conducted in Cambodia, Indonesia, and the US. In addition to our previous study on the distribution of *tfs*/cagPAI in Indonesia, we conducted the same analysis of whole-genome contigs of Cambodia and US strains (Table S6). The prevalence of cagPAI in Vietnam strains (94%) was highest among strains from 4 countries and significant compared to the prevalence of cagPAI in US (81%, *P* = 0.01) and Indonesian (55%, *P* < 0.0001) strains but not significant compared to the prevalence of Cambodian strains (91%) (Fig. 4). In contrast, the prevalence of *tfs3* in Vietnam strains (46%) was not statistically different from that in the US (58%), Indonesia (41%), and Cambodia (45%) strains. The prevalence of *ctkA* in Vietnam strains was statistically higher than that in the US strains (29% vs 2%, *P* = 0.0002).

Although the prevalence of the *dupA* cluster in the Vietnam and Cambodia strains tended to be lower than that in the US strains (15% vs 23%, *P* = 0.22), the prevalence of the *dupA* cluster in strains in these three countries was higher than that in Indonesia strains (all *P* < 0.05). The prevalence of *dupA*( +) in the US strains (60%) was higher compared to those of Vietnam (15%), Indonesian (5%), and Cambodian (19%) strains (all *P* < 0.0001). However, 37% of the US strains possessed the short-form *dupA* (78% in coverage compared to long-form *dupA*) (Table S6); *dupA* was in the long form in Vietnam and Cambodia strains (Table 4).

## Discussion

In this study, the prevalence of strains possessing the *tfs* ICE was high among Vietnam strains (90.4%), which were divided into *tfs3* ICE (45%) and *tfs4* ICE (77%) (Table S6). The distribution of *tfs* ICE was dependent on the ethnic and geographical phylogeny of strains^14,15^. This could explain the difference in *tfs* ICE from that observed in our previous study in Indonesia (54.3%), in which the strains were isolated from several ethnicities^15^; all strains in this study were isolated from people of the Kinh ethnicity in Vietnam and predominantly belonged to the hspEAsia population (95.5%) (Fig. 3). The pooled prevalence of *tfs* ICE in *H. pylori* samples selected from seven distinct phylogeographic populations was 92.8% for *tfs4* ICE and 62.2% for *tfs3* ICE^14^. In addition, the distributions of *tfs3* and *tfs4* in hpEurope/hpAfrica1 strains (139 strains) were 69% and 96%, respectively, and were different from those of the hspEAsia strains (28 strains): 53% and 71%, respectively^14^. Additionally, although there were a few whole-genome hpAfrica2 strains available, all possessed *tfs4c* (L2C2R2) (100%)^14^. As shown in Tables S6 and S7, the prevalences of *tfs3* and *tfs4* were 43% and 76%, respectively, in our hspEAsia strains, which is consistent with the results of previous studies. Furthermore, the prevalence of *tfs* ICE in our previous study in Indonesia, which was composed of hpNEAfrica/hpEurope/hpAsia2 populations, was significantly lower than that in Vietnam (54.3% vs 90.4%, *P* < 0.001), also supporting the different prevalence of *tfs* ICE between populations^15^.

*dupA*, a *virB4* homolog of *tfs4b*, was considered to be a marker of DU, but was not universally associated with disease development^29,30^. Our previous study indicated that the presence of *dupA* and its neighboring *vir* genes, which form the complete *tfs4b* cluster (or *dupA* cluster), is considered to be a more reliable disease-marker compared with single *dupA*^19,20^*.* Vietnam strains possessed a low prevalence of *dupA* (15.4%) compared to those from the US (69.3%), Japan (37%), Korea (37%), and Columbia (55%)^18,19^. In this study, the prevalence of the *dupA* cluster in Vietnam strains was significantly lower than that in the US strains (15.4% vs 69.3%, *P* < 0.0001)^19^ but was slightly higher than that in the Indonesia strains^15^ (15.4% vs 5%, *P* < 0.01); Indonesia has a low risk of gastric cancer. Moreover, there was a remarkably low incidence of *H. pylori*-related disease in the Nigerian population (hspWAfrica), which possesses a high prevalence of the truncated R1 module of *tfs4b* (L1/L2C1R1f.), in which is a short-form *dupA* with a lack of *virB2* and *virB3*; although this population contained the high prevalences of *cagA* and *vacA s1m1* genotype^31^. In our analysis, most of the US strains (hspWAfrica) similarly possessed L1/L2C1R1f., which supported the role of this *tfs4* subtype in hspWAfrica1 strains. In addition, our previous study in Okinawa (Japan) showed that long-form and intact long-form *dupA* increased the risk of gastroduodenal diseases (gastric ulcer and gastric cancer) but not cagA^26^. These studies suggested that the deficient of *dupA* cluster would be reversibly associated with the severity of diseases and have a protective role in some settings. The function of *dupA* cluster has not yet been determined, but it might support the translocation of effector protein(s) similarly to CagA injection by the cagPAI. In our study, only 21/136 strains (15.4%) had the *dupA,* and 11 of them (8.1%) were non-functional *dupA* caused by mutation (frameshift, premature stop codon, and deletion). In addition, non-intact *dupA* was more frequently observed in CG (15.3%) patients than in NCGC patients (7.8%), while there was a negative correlation between *dupA* and DU patients. Our data suggested that there was an effect of *dupA* on pathogenesis, despite its low prevalence in Vietnam. The *dupA* gene encodes homologs of *VirB4* ATPase. The Pfam search shows that *dupA* contains the CagE_TrbE_VirB domain and FtsK/SpoIIIE family. The FtsK/SpoIIIE domain contains a putative ATP- binding P-loop motif, is involved in cell division and peptidoglycan synthesis or modification and is implicated in intracellular chromosomal DNA transfer. Members of the TraG/TraD family are potential NTP hydrolases that are essential for DNA processing and the mating pair formation system. The in vitro study showed that there was a more positive correlation between IL-8 expression and *dupA*-positive strains than *dupA*-negative strains^18^.

The prevalence of *ctkA* (*jhp0940* in strain J99), a cell-translocating kinase, was 41.2% in gastric cancer patients but zero among the strains isolated from patients with gastritis (*P* < 0.0006) in Costa Rica and was the first disease-marker of *tfs3* ICE^32^. In addition, *ctkA* is located within the *tfs3* ICE and is variably distributed in diverse *H. pylori* strains^33^. There was evidence that the *tfs3* T4SS supported and increased the pro-inflammatory activity of effector protein (CtkA) in gastric epithelial cells, and it is suggested that *tfs3* may form a novel T4SS assembly for protein secretion similar to cagPAI^33^. However, there was no association between *ctkA* alone or in combination with complete *tfs3* with the severity of clinical outcomes in Vietnam. Compared to the US strains, the high prevalence of *ctkA* in Vietnam strains might indicate population-dependent variation between geographical regions. Our previous study in Indonesia revealed that intact cagPAI/*tfs* ICE-positive strains had significantly higher antral activity than non-intact cagPAI/*tfs*-negative and non-intact cagPAI/*tfs*-positive strains; no difference was observed between intact cagPAI/*tfs*-negative and non-intact cagPAI/*tfs*-negative^15^. In this study, the prevalences of complete cagPAI/incomplete *tfs3* as well as complete cagPAI/complete *tfs3* were 24.3% and 15.4%, respectively, which implied that functional cagPAI, regardless of *tfs3* status, correlated with disease progression. These results indicate that cagPAI is the factor most affecting the development of gastroduodenal diseases, and its combination with other virulence factors, such as *tfs3/tfs4*, might increase the severity of clinical outcomes. The role of *tfs* ICE in pathogenesis could be associated with inflammation induction in gastric epithelial cells^15^, because T4SS encoded by this element promotes IL-8 expression independent of the presence of cagPAI^12^ and was reinforced when *tfs* ICE was present^20^. Although the association of the intact *dupA* cluster with clinical outcomes had been determined, the association of *tfs3* ICE with gastroduodenal diseases is still not yet determined. In our study, there was a dominant complete *tfs3* ICE in DU patients, which was the first report of *tfs3* ICE in diseases. In the future, more studies about the prevalence and association of *tfs3/4* ICE and cagPAI with clinical outcomes need to be conducted to clarify the role of *tfs3* ICE in gastroduodenal diseases.

Although the biology of *tfs* ICE in *H. pylori* is not yet well understood, *tfs* ICE might have an impact on the fitness benefit, which helps these bacteria adapt to the gastric environment^11^. There is a long and complex history of acquisition, module exchange, and rearrangement of *tfs* ICEs within various *H. pylori* populations^13,14^. A limitation of our study was the unfinished, whole draft genome, which was fragmented into several contigs. Because of the fragmentary nature of the draft genome, it has remained refractory to the study of long chromosomal segments such as *tfs* ICE. It is necessary to utilize both the Illumina sequencer and the 3^rd^ generation sequencer (PacBio, Nanopore), which generates a full-genome contig, to study the structure and location of *tfs* ICE in the *H. pylori* genome^34^. Secondly, our study was based on the comparison between the DNA sequences, and the expression of the T4SS gene cluster of *tfs* ICE at the mRNA/protein level has not yet been determined.

In summary, the acquisition of *tfs3/4* ICE was common in *H. pylori* strains isolated from patients with gastroduodenal disease in Vietnam, and the complete cluster of *tfs3* ICE was a reliable marker for the severity of diseases in the infected population.

## Methods

### Sample collection

A total of 136 *H. pylori* strains were isolated from patients with gastroduodenal diseases in Ha Noi and Ho Chi Minh, Vietnam from 2009–2017. *H. pylori* culture was performed as previously described^22^. The 109 strains were used in our previous epidemiological studies^21,22,27^ strains were additionally cultured in this study. All strains were isolated from patients with Kinh ethnicity and were divided into 3 disease-groups: NCGC (51 strains), DU (46 strains), and CG (39 strains). These strains had never been evaluated before to assess the status of cagPAI and *tfs* ICE*.* Local ethics approval was obtained from the Ethics Committee of Cho Ray Hospital and 108 Military Hospital, and written informed consent was obtained from all patients. The study was also approved by the Ethics Committee of Oita University Faculty of Medicine, Japan and was carried out in accordance with the Declaration of Helsinki (https://doi.org/10.1515/9783110208856.233).

### DNA preparation and next-generation sequencing

The total genomic DNA of isolates were extracted using the QIAamp DNA Mini Kit (QIAGEN, UK) and quantified by Quantus Fluorometer (Promega Corporation). The DNA library was prepared using the Nextera XT DNA sample kit (Illumina, San Diego, CA, USA) allowing paired-end sequencing techniques. Short-read sequences of *H. pylori* were obtained from next-generation sequencing (NGS); Hiseq and Miseq platform (Illumina, Inc., San Diego, CA, USA).

### Software tools for *H. pylori* genome analysis

The quality of raw sequencing reads was checked with FASTQC (Babraham Bioinformatics)^35^, filtered, and low-quality bases trimmed (< Q30) using Trimmomatic^36^. Trimmed paired-read sequences were subsequently de novo assembled to generate contigs using Shovill assembly^35^ (https://github.com/tseemann/shovill). The quality of de novo assembly included: contig numbers; total length; and N50, N75, L50, L75, and GC percentage, and the completeness of whole-genome contigs was checked by QUAST^37^ and BUSCO^37^. Pairwise sequence alignment was performed for sequence comparison between *tfs3* ICE (strain Gambia94/24 and strain India7), *tfs4a* ICE (strain P12), *tfs4b* ICE (strain Shi470), *tfs4c* ICE (strain R036d), cagPAI (strain 26,695), and comB (strain 26,695) (https://www.ebi.ac.uk/Tools/psa/)13, which was used to make custom databases. To detect the presence of *tfs* ICE, cagPAI, and comB in each strain, assembled contigs were analyzed by ABRICATE (https://github.com/tseemann/abricate), a pipeline for determining virulence factor with the user’s custom databases.

### Genotyping and phylogenetic analysis

Phylogeny and population assignment were constructed based on 7 house-keeping genes (*atpA*, *efp*, *trpC*, *ppa*, *mutY*, *yphC,* and *ureI*). Each gene sequence was retrieved and concatenated from assembled contig into FASTA format by the MLSTcheck package (https://github.com/sanger-pathogens/mlst_check/). Additionally, a total of 379 *H. pylori* strains with known origins available at PubMLST (http://pubmlst.org/helicobacter/), originally described by Falush et al.^38^, were included in the analysis. Sequences were aligned using MAFFT alignment algorithm^39^. The Newick tree format was generated using the neighbor-joining algorithm (Kimura-2 method) of MEGA software version 7^40^, and a phylogenetic tree was constructed using the same software.

### Statistical method

Fisher’s exact test and proportion test were used. A logistic regression model was used to calculate the odds ratio (OR) and 95% confidence interval (CI) between virulence factors and clinical outcomes. For all statistical tests, *P* values < 0.05 were accepted as statistically significant. Data analysis was performed with RStudio v1.1.4 (RStudio, Inc, USA).

## Supplementary Information

Supplementary Information 1.

Supplementary Information 2.
