# Supplementary material for: Helicobacter pylori type 4 secretion systems as gastroduodenal disease markers
Source: Sci Rep. 2021 Feb 25;11:4584. doi: 10.1038/s41598-021-83862-1 (PMC7907105; doi:10.1038/s41598-021-83862-1)
Supplement: Supplementary file 2 — Supplementary Information 2. [file 41598_2021_83862_MOESM2_ESM.pdf]

Helicobacter pylori type 4 secretion systems as gastroduodenal disease markers

Bui Hoang Phuc, Vo Phuoc Tuan, Ho Dang Quy Dung, Tran Thanh Binh, Pham Huu Tung, Tran Dinh Tri, Ngo Phuong Minh Thuan, Vu Van Khien, Tran Thi Huyen Trang, Junko Akada, Takeshi Matsumoto, and Yoshio Yamaoka

Supplementary Material

Table S1: Assembly quality of 136 strains in this study

Strain name	Number of contigs[a]	Largest comtigs	Total length[b]	N50	N75	L50	L75	GC%	BUSCO	Accession
VN0211	28	337,040	1,630,376	154,782	59,734	4	9	38.67	88.5	BNNC01000001-BNNC01000034
VN0212	33	237,579	1,616,411	104,300	52,696	5	11	38.7	87.8	BNND01000001-BNND01000037
VN0219	32	434,738	1,686,404	218,516	57,859	3	7	38.57	87.2	BNNE01000001-BNNE01000044
VN0220	46	236,982	1,605,297	68,650	46,465	7	14	38.78	87.8	BNNF01000001-BNNF01000054
VN0223	30	330,397	1,582,744	167,078	74,463	3	7	38.85	87.8	BNNG01000001-BNNG01000031
VN0224	29	362,285	1,578,831	107,267	55,467	4	10	38.83	87.8	BNNH01000001-BNNH01000030
VN0227	39	297,830	1,588,929	106,597	53,568	5	11	38.73	88.5	BNNI01000001-BNNI01000041
VN0228	31	301,343	1,575,300	130,353	56,015	4	10	38.8	88.5	BNNJ01000001-BNNJ01000034
VN0229	42	447,435	1,660,119	87,401	53,729	4	10	38.6	87.2	BNNK01000001-BNNK01000052
VN0232	54	334,509	1,624,318	76,726	36,305	6	14	38.69	87.8	BNNL01000001-BNNL01000062
VN0235	25	430,471	1,567,205	144,419	70,040	3	7	38.85	87.2	BNNM01000001-BNNM01000025
VN0240	39	316,923	1,583,113	55,242	46,686	7	14	38.8	88.5	BNNN01000001-BNNN01000049
VN0244	37	288,499	1,612,297	75,303	52,933	5	12	38.12	87.2	BNNO01000001-BNNO01000044
VN0246	38	234,831	1,594,478	98,202	50,592	6	12	38.76	85.8	BNNP01000001-BNNP01000043
VN0264	38	217,643	1,628,795	79,438	51,971	6	13	38.68	87.8	BNNQ01000001-BNNQ01000046
VN0268	29	336,628	1,609,558	108,256	86,304	4	8	38.72	87.8	BNNR01000001-BNNR01000033
VN0271	34	676,484	1,625,401	184,705	53,219	2	7	38.72	87.8	BNNS01000001-BNNS01000038
VN0272	29	267,305	1,573,025	104,680	51,603	5	10	38.84	86.5	BNNT01000001-BNNT01000030
VN0274	46	336,085	1,580,312	72,729	46,828	4	11	38.81	87.2	BNNU01000001-BNNU01000053
VN0280	44	170,803	1,589,529	65,503	38,085	8	16	38.78	88.5	BNNV01000001-BNNV01000046
VN0283	46	129,057	1,603,717	53,616	39,968	10	18	38.79	88.5	BNNW01000001-BNNW01000058
VN0324	29	429,620	1,577,639	177,684	59,999	3	8	38.85	88.5	BNNX01000001-BNNX01000030
VN0338	43	234,055	1,642,070	109,182	51,935	6	12	38.64	87.8	BNNY01000001-BNNY01000049
VN0348	36	246,525	1,644,511	98,916	60,623	5	11	38.83	89.9	BNNZ01000001-BNNZ01000037
VN0352	23	370,752	1,586,424	211,641	68,205	3	7	38.83	87.2	BNOA01000001-BNOA01000028
VN0355	31	655,895	1,606,763	108,916	60,114	3	8	38.67	87.2	BNOB01000001-BNOB01000033
VN0361	45	321,825	1,637,587	82,853	53,790	5	11	38.66	87.8	BNOC01000001-BNOC01000054
VN0363	28	541,182	1,579,593	136,137	57,996	3	8	38.86	88.5	BNOD01000001-BNOD01000032
VN0366	35	330,120	1,573,206	107,419	65,919	4	8	38.8	87.8	BNOE01000001-BNOE01000036
VN0382	30	230,640	1,566,005	172,755	53,759	4	8	38.83	88.5	BNOF01000001-BNOF01000030
VN0390	29	432,180	1,574,742	124,862	53,167	3	10	38.8	87.8	BNOG01000001-BNOG01000037
VN0401	31	238,985	1,641,573	106,492	59,577	6	12	38.64	87.8	BNOH01000001-BNOH01000032
VN0403	33	232,881	1,559,731	66,989	54,639	7	13	38.87	88.5	BNOI01000001-BNOI01000035
VN0405	39	235,478	1,613,669	68,732	42,741	6	13	38.7	87.8	BNOJ01000001-BNOJ01000050
VN0406	27	648,464	1,573,126	178,717	58,002	2	7	38.77	87.8	BNOK01000001-BNOK01000027
VN0410	31	131,312	1,599,970	86,876	59,341	8	13	38.73	87.8	BNOL01000001-BNOL01000033

VN0411	30	325,552	1,566,442	131,082	56,340	4	9	38.86	88.5	BNOM01000001-BNOM01000032
VN0418	30	235,227	1,668,999	172,176	77,198	4	8	38.6	87.8	BNON01000001-BNON01000030
VN0420	42	233,976	1,630,302	91,879	51,161	6	12	38.67	86.5	BNOO01000001-BNOO01000047
VN0431	32	423,191	1,566,026	108,091	55,116	4	9	38.84	88.5	BNOP01000001-BNOP01000033
VN0434	33	235,259	1,597,570	104,183	59,727	6	11	38.7	87.2	BNOQ01000001-BNOQ01000035
VN0438	25	362,194	1,571,559	199,187	58,533	3	8	38.83	87.8	BNOR01000001-BNOR01000026
VN0443	97	227,431	1,596,903	56,849	24,308	9	20	38.76	87.2	BNOS01000001-BNOS01000127
VN0448	41	334,954	1,600,753	107,589	42,908	5	13	38.71	87.2	BNOT01000001-BNOT01000047
VN0452	42	333,290	1,557,214	75,901	38,013	5	13	38.9	87.8	BNOU01000001-BNOU01000059
VN0453	61	237,246	1,595,496	65,116	42,898	8	16	38.78	85.8	BNOV01000001-BNOV01000082
VN0464	35	227,290	1,569,209	122,833	63,671	5	9	38.8	87.8	BNOW01000001-BNOW01000041
VN0472	39	400,244	1,599,160	67,410	52,635	5	12	38.75	87.8	BNOX01000001-BNOX01000046
VN0481	33	692,031	1,593,779	108,683	54,198	2	8	38.74	87.8	BNOY01000001-BNOY01000036
VN0484	129	100,573	1,606,762	45,923	20,959	12	25	38.84	87.9	BNOZ01000001-BNOZ01000173
VN0495	38	237,963	1,605,575	95,768	60,913	6	11	38.78	87.2	BNPA01000001-BNPA01000046
VN0511	40	393,223	1,643,921	207,635	67,493	3	7	38.66	88.5	BNPB01000001-BNPB01000047
VN0559	43	236,649	1,651,698	108,790	54,866	5	10	38.67	87.8	BNPC01000001-BNPC01000046
VN0754	31	318,655	1,605,059	95,059	65,816	5	10	38.74	87.2	BNPK01000001-BNPK01000036
VN0759	39	240,312	1,589,900	72,919	48,362	7	14	38.81	87.8	BNPL01000001-BNPL01000044
VN1154	29	313,196	1,573,179	107,557	65,489	4	9	38.77	89.2	BNPQ01000001-BNPQ01000031
VN1155	33	378,093	1,631,053	108,992	54,231	5	10	38.63	87.8	BNPR01000001-BNPR01000036
VN1156	30	231,954	1,574,628	70,526	58,237	5	11	38.84	87.8	BNPS01000001-BNPS01000034
VN1158	45	142,847	1,634,749	65,407	48,152	9	16	38.61	88.5	BNPT01000001-BNPT01000050
VN1161	36	242,737	1,585,941	74,033	53,617	6	12	38.73	87.2	BNPU01000001-BNPU01000045
VN1163	31	333,340	1,628,992	107,339	53,438	4	10	38.67	87.8	BNPV01000001-BNPV01000032
VN1165	33	459,318	1,595,474	111,244	54,119	3	10	38.68	87.8	BNPW01000001-BNPW01000034
VN1167	38	287,839	1,567,728	76,147	52,820	6	12	38.76	87.2	BNPX01000001-BNPX01000043
VN1169	41	234,189	1,607,416	72,653	48,808	6	13	38.67	88.5	BNPY01000001-BNPY01000045
VN1171	44	149,660	1,589,189	59,055	35,599	9	17	38.88	86.5	BNPZ01000001-BNPZ01000046
VN1172	53	173,717	1,645,458	77,084	38,972	8	16	38.91	87.2	BNQA01000001-BNQA01000059
VN1175	36	167,457	1,567,123	75,357	48,366	7	14	38.85	87.2	BNQB01000001-BNQB01000037
VN1176	56	111,892	1,568,799	59,099	36,227	10	18	38.89	88.5	BNQC01000001-BNQC01000068
VN1178	55	235,774	1,573,500	67,356	39,794	7	15	38.88	86.5	BNQD01000001-BNQD01000059
VN1180	38	467,285	1,621,371	74,667	52,428	5	12	38.73	86.5	BNQE01000001-BNQE01000041
VN1183	35	333,906	1,601,032	77,353	38,210	5	11	38.74	87.8	BNQF01000001-BNQF01000043
VN1184	42	226,656	1,683,321	109,600	60,695	5	11	38.69	89.2	BNQG01000001-BNQG01000049
VN1186	52	231,167	1,553,586	54,086	38,185	7	15	38.91	87.2	BNQH01000001-BNQH01000068
VN1192	37	246,754	1,625,927	76,918	46,809	6	13	38.75	87.8	BNQI01000001-BNQI01000051

VN1193	33	356,367	1,569,529	68,996	52,407	6	12	38.83	87.2	BNQJ01000001-BNQJ01000038
VN1195	46	152,432	1,570,491	56,539	33,752	9	17	38.84	88.5	BNQK01000001-BNQK01000055
VN1196	32	232,509	1,607,485	101,854	55,504	5	11	38.68	87.8	BNQL01000001-BNQL01000037
VN1197	25	407,899	1,576,234	163,670	54,775	3	7	38.84	87.2	BNQM01000001-BNQM01000027
VN1199	53	143,730	1,552,412	59,597	33,798	9	18	39.01	89.9	BNQN01000001-BNQN01000062
VN1200	42	209,434	1,643,572	86,665	52,865	6	12	38.9	87.2	BNQO01000001-BNQO01000052
VN1202	40	289,939	1,577,348	68,022	44,967	6	13	38.88	88.5	BNQP01000001-BNQP01000046
VN1203	33	261,369	1,560,976	122,661	57,430	4	10	38.88	89.2	BNQQ01000001-BNQQ01000037
VN1204	49	182,970	1,632,040	81,209	51,237	7	13	38.72	88.5	BNQR01000001-BNQR01000055
VN1205	33	321,209	1,562,120	84,335	52,122	6	12	38.9	87.8	BNQS01000001-BNQS01000038
VN1210	45	276,005	1,593,975	66,469	39,206	7	15	38.8	87.8	BNQT01000001-BNQT01000053
VN1212	27	398,794	1,604,961	171,487	60,010	3	8	38.66	88.5	BNQU01000001-BNQU01000028
VN1213	39	178,751	1,570,582	88,158	53,625	7	13	38.85	87.2	BNQV01000001-BNQV01000043
VN1215	56	144,923	1,607,869	48,963	26,712	12	22	38.75	87.2	BNQW01000001-BNQW01000069
VN1217	33	240,457	1,568,845	105,419	55,487	5	11	38.82	89.2	BNQX01000001-BNQX01000040
VN1218	28	280,854	1,560,660	149,567	69,590	4	8	38.92	88.5	BNQY01000001-BNQY01000030
VN1219	32	332,612	1,570,697	106,558	65,316	4	9	38.82	88.5	BNQZ01000001-BNQZ01000034
VN1221	33	323,707	1,600,328	109,285	55,421	4	10	38.73	87.2	BNRA01000001-BNRA01000038
VN1222	56	186,146	1,599,128	48,846	34,306	11	21	38.75	87.8	BNRB01000001-BNRB01000061
VN1224	26	276,011	1,616,667	191,015	57,526	4	8	38.68	88.5	BNRC01000001-BNRC01000030
VN1225	38	189,394	1,598,103	68,942	50,912	6	13	38.74	87.8	BNRD01000001-BNRD01000046
VN1226	35	234,276	1,553,258	104,260	55,437	6	11	38.89	88.5	BNRE01000001-BNRE01000039
VN1227	33	180,950	1,555,386	60,109	44,050	8	15	38.88	87.8	BNRF01000001-BNRF01000037
VN1229	40	303,291	1,646,110	98,179	52,866	6	12	38.9	87.8	BNRG01000001-BNRG01000041
VN1230	47	141,656	1,554,919	54,711	38,825	11	19	38.91	87.8	BNRH01000001-BNRH01000052
VN1233	57	230,695	1,569,932	43,902	32,171	11	22	38.78	85.8	BNRI01000001-BNRI01000063
VN1235	42	225,514	1,614,913	75,785	50,743	7	14	38.72	87.8	BNRJ01000001-BNRJ01000048
VN1236	36	246,849	1,579,799	73,711	52,588	6	12	38.82	87.2	BNRK01000001-BNRK01000038
VN1237	33	308,768	1,595,658	99,493	60,995	5	10	38.76	88.5	BNRL01000001-BNRL01000042
VN1239	37	240,237	1,595,872	73,587	52,944	6	12	38.74	87.8	BNRM01000001-BNRM01000041
VN1240	59	172,603	1,572,062	61,599	30,075	9	18	38.85	86.5	BNRN01000001-BNRN01000087
VN1241	39	235,168	1,620,787	82,821	44,758	6	13	38.67	87.2	BNRO01000001-BNRO01000044
VN1244	53	233,128	1,628,617	61,073	38,865	8	16	38.75	87.8	BNRP01000001-BNRP01000061
VN1246	53	185,606	1,592,059	52,248	32,198	10	19	38.76	88.5	BNRQ01000001-BNRQ01000065
VN1247	36	176,140	1,576,733	61,302	48,389	7	14	38.83	86.5	BNRR01000001-BNRR01000041
VN1248	52	220,604	1,559,161	59,431	34,472	7	16	38.85	88.5	BNRS01000001-BNRS01000064
VN1249	37	235,148	1,617,478	75,863	48,228	6	13	38.77	87.8	BNRT01000001-BNRT01000039
VN1250	28	428,071	1,573,970	107,165	57,261	4	10	38.85	89.2	BNRU01000001-BNRU01000028

VN1251	41	244,456	1,599,681	68,086	51,992	7	14	38.76	87.2	BNRV01000001-BNRV01000049
VN1258	23	675,092	1,587,516	174,398	53,829	2	7	38.78	87.8	BNRW01000001-BNRW01000026
VN1260	26	366,882	1,619,970	217,986	54,520	3	8	38.79	87.8	BNRX01000001-BNRX01000027
VN1261	29	436,746	1,647,578	107,386	55,364	4	9	38.62	87.8	BNRY01000001-BNRY01000032
VN1262	48	319,039	1,678,279	59,352	49,294	6	14	38.58	88.5	BNRZ01000001-BNRZ01000057
VN1263	30	433,970	1,618,364	170,034	69,125	3	7	38.68	88.5	BNSA01000001-BNSA01000031
VN1264	35	532,693	1,621,926	130,061	63,467	3	7	38.65	87.8	BNSB01000001-BNSB01000039
VN1266	26	371,650	1,607,715	206,830	64,795	3	7	38.71	88.5	BNSC01000001-BNSC01000027
VN1269	36	414,614	1,592,773	96,596	52,491	4	10	38.81	87.8	BNSD01000001-BNSD01000038
VN1270	34	235,450	1,606,632	133,890	51,325	5	10	38.76	87.2	BNSE01000001-BNSE01000035
VN1272	28	457,595	1,603,253	75,237	59,153	4	10	38.74	87.2	BNSF01000001-BNSF01000033
VN1274	33	235,483	1,587,600	104,717	59,526	5	10	38.77	88.5	BNSG01000001-BNSG01000036
VN1276	30	211,262	1,617,262	131,410	57,339	5	10	38.69	87.2	BNSH01000001-BNSH01000034
VN1277	43	246,971	1,570,223	76,213	37,505	7	15	38.85	87.2	BNSI01000001-BNSI01000047
VN1279	35	327,671	1,601,596	91,715	52,380	5	11	38.73	87.8	BNSJ01000001-BNSJ01000038
VN1280	27	434,171	1,583,722	156,037	55,363	3	9	38.86	87.2	BNSK01000001-BNSK01000029
VN1281	34	236,739	1,577,605	166,231	52,896	4	10	38.86	87.2	BNSL01000001-BNSL01000038
VN1283	64	138,799	1,611,849	47,867	30,988	11	21	38.68	87.2	BNSM01000001-BNSM01000077
VN1284	44	147,729	1,567,670	56,835	38,441	10	18	38.84	87.2	BNSN01000001-BNSN01000057
VN1286	62	117,495	1,629,874	61,406	35,038	10	19	38.66	87.8	BNSO01000001-BNSO01000074
VN1288	49	216,086	1,590,292	53,358	36,077	10	18	38.69	88.5	BNSP01000001-BNSP01000052
VN1289	49	204,476	1,598,138	50,184	28,401	8	18	38.8	87.8	BNSQ01000001-BNSQ01000052
VN1290	54	233,200	1,606,787	53,732	31,829	7	16	38.71	87.2	BNSR01000001-BNSR01000063
VN1291	81	201,587	1,672,214	47,413	26,793	10	23	38.57	87.8	BNSS01000001-BNSS01000093

a: The number of contigs (≥500 bp)

b: Total length of contigs (≥500 bp)

Table S2: Annotation of *tfs* ICE genes in *H. pylori* genome

Annotation	*tfs3* (India7)	*tfs3* (Gambia94/24)			*tfs4a* (P12)	*tfs4b* (Shi470)	*tfs4c* (R036d)
xerT	3725	7345	***tfs4* module**	**L**	437	4480	1010
virB6	3760	7375			439	4485	1008
virD2	3720	7340		**C**	451	4530	996
virD4	3825	7455			454	4545	993
virB11	3835	7465			458	4565	990
virB10	3855	7485			462	4590	986
virB9	3860	7490			463	4595	985
virB8	3865	7495		**R**	464	4600	984
virB7	3870	7500			465	4605	982
virB4	3875	7505			467	4615	980
virB3	3885	7515			468	4620	979
virB2	3890	7520			469	4625	978

Table S3: Pairwise sequence comparison of T4SS nucleotide sequences

Percentage sequence identity of nucleotide sequence from pairwise sequence comparison of *vir* homologous genes

**T4SS proteins**	***A. tumefaciencs* pTi plasmid[1] vs**				**comB[2] vs**				**cagPAI[2] vs**			
	***tfs3* (Gambia94/24)**	***tfs4a* (P12)**	***tfs4b* (Shi470)**	***tfs4c* (R036d)**	***tfs3* (Gambia94/24)**	***tfs4a* (P12)**	***tfs4b* (Shi470)**	***tfs4c* (R036d)**	***tfs3* (Gambia94/24)**	***tfs4a* (P12)**	***tfs4b* (Shi470)**	***tfs4c* (R036d)**
**VirB2**	38.5	41.9	42.2	35.6	61.0	47.6	47.4	46.9	45.3	41.8	45.5	39.9
**VirB3**	40.6	42.1	42.5	21.4	57.6	52.0	59.6	47.4	-	-	-	-
**VirB4**	43.6	45.5	43	44.8	56.5	52.3	53.2	49.8	46.2	45.8	45.7	45.5
**VirB6**	40.1	38.3	39.4	38.7	-	44.4	-	44.3	44.4	42.6	44.3	47.2
**VirB7**	44.2	33.7	42.7	-	50.7	54.6	53.5	-	<15	<15	<15	-
**VirB8**	37.7	35.5	41.6	42.2	42.3	37.9	43.1	49.2	44.4	38.4	44.1	48.1
**VirB9**	29.6	34.4	34.9	34.9	39.3	40.5	40.2	40.6	43.2	46.1	46.9	46.8
**VirB10**	42.5	42.3	43.2	42.1	59.9	54.2	53.9	53.1	38.4	43.2	41.6	37.9
**VirB11**	43.6	45.4	45.2	43.5	-	-	-	-	50.9	46.9	45	48.5
**VirD2**	-	-	-	-	-	-	-	-	-	-	-	-
**VirD4**	-	-	-	-	-	-	-	-	42.6	40.2	38.5	46.4

1: T4SS genes of *Agrobacterium tumerfaciens* pTi plasmid strain C58

2: indicated cagPAI, comB and *tfs3/4a/4b/4c* were retrieved from the same strain background

Table S4: Pairwise sequence comparison of T4SS nucleotide sequences of *tfs3, tfs4a, tfs4b, tfs4c* ICE

tfs ICE genes	***tfs3* (Gambia94/24) vs**				***tfs4b* (Shi470) vs**		***tfs4a* (P12) vs**
	***tfs3* (India7)**	***tfs4a* (P12)**	***tfs4b* (Shi470)**	***tfs4c* (R036d)**	***tfs4a* (P12)**	***tfs4c* (R036d)**	
xerT	**91.2**	68	61.2	67.6	62.1	62.1	**98.2**
virB2	**91.6**	51.2	48.8	50.8	55.3	49.5	**83.5**
virB3	**89**	56.1	56.7	56.3	59.8	56.4	**96.3**
virB4	**91**	53.5	54.7	53.7	56.5	55.2	**96.5**
virB6	66.4	46.2	47.7	47.1	51.9	52.1	**93.7**
virB7	70.9	48.3	52.4	n.a	56.5	n.a	n.a
virB8	**86.8**	52.6	52.9	52.1	59	58.5	**96.3**
virB9	**83.3**	45.4	46.3	45.8	**95.8**	**96.3**	**96.5**
virB10	**91**	57.2	59.1	57.5	**82.5**	**82.3**	**98**
virB11	**93.9**	58.3	58.3	56.2	**98.4**	61.6	62
virD2	**87.9**	49.4	48.8	49.8	**95.5**	54.4	55.1
virD4	**82.6**	46.1	45.7	54.1	**98.8**	48.7	48.5

Table S5: Diversity of T4SS sequences of *tfs3*, *tfs4a/b/c*, cagPAI, and comB of H. pylori strains isolated from Vietnam

tfs ICE type (total number of Vietnam strains possessed)		T4SS genes	No. of strains possessing the corresponding *vir* gene with 80%< identity	Range of nucleotide identity to reference T4SS (%)	Reference strain
***tfs3* (n=62)**		*xerT*	59	91–99	Gambia94/24 and India7
		virB2	37	91–98	
		virB3	37	88–93	
		virB4	37	89–97	
		virB6	46	85–99	
		virB7	32	96–98	
		virB8	32	82–98	
		virB9	35	86–97	
		virB10	35	88–94	
		virB11	35	91–95	
		virD4	35	81–86	
		virD2	45	86–97	
***tfs4* (n=105)**	**L1 (n=41)**	*xerT*	41	96-98	Shi470, P12, R036d
		virB6	41	96-98	
	**L2 (n=64)**	*xerT*	64	95-98	
		virB6	28	94-97	
	**C1 (n=40)**	*virD2*	40	92-100	
		virD4	30	97-98	
		virB11	25	94-99	
		virB10	27	80-98	
		virB9	27	92-98	
	**C2 (n=4)**	*virD2*	4	96-97	
		virD4	4	97-99	
		virB11	4	98	
		virB10	4	96	
		virB9	4	94-97	
	**R1 (n=27)**	*virB8*	26	97-100	
		virB7	27	92-100	
		virB4	21	96-99	
		virB3	21	98-100	
		virB2	21	97-99	
	**R2 (n=38)**	*virB8*	5	97-98	
		virB7	0		
		virB4	38	97-98	
		virB3	38	91-99	
		virB2	38	94-99	
**cagPAI (n=130)**		*virB2*	129	93–98	26695
		virB4	128	98–99	
		virB5	128	93–96	
		virB6	129	97–99	
		virB7	129	98–99	
		virB8	128	98–99	
		virB9	129	97–99	
		virB10	129	98	
		virB11	130	99	
		virD4	130	98	
***comB* (n=136)**		*virB2*	136	97–100	26695
		virB3	136	95–97	
		virB4	136	97	
		virB7	136	91–100	
		virB8	136	98	
		virB9	136	92–97	
		virB10	136	98	

Table S6: *tfs3/tfs4* ICEs in *H. pylori* strains isolated in Vietnam

Vietnam strains	Country	Diseases[e]	Population[f]	cagPAI	*tfs* classification[g]	*tfs3* ICE[c]	*tfs4* ICE[d]	Module of *tfs4* ICE
VN0211	Vietnam	NCGC	hspEAsia	complete	3 and 4	complete	incomplete	L2
VN0212	Vietnam	NCGC	hspEAsia	complete	3 and 4	incomplete	incomplete	L2
VN0219	Vietnam	NCGC	hspEAsia	complete	3 and 4	complete	incomplete	L1C1
VN0220	Vietnam	NCGC	hspEAsia	complete	4		incomplete	L2R2
VN0223	Vietnam	NCGC	hspEAsia	complete	4		incomplete	L2R2
VN0224	Vietnam	NCGC	hspEAsia	complete	3 and 4	incomplete	incomplete	L2
VN0227	Vietnam	NCGC	hspEAsia	complete	4b		incomplete	L1C1
VN0228	Vietnam	NCGC	hspEAsia	complete	4		incomplete	L2R2
VN0229	Vietnam	NCGC	hspEAsia	complete	3 and 4	complete	incomplete	L2
VN0232	Vietnam	NCGC	hspEAsia	complete	4b		complete	L1C1R1
VN0235	Vietnam	NCGC	hspEAsia	complete	4		incomplete	L1
VN0240	Vietnam	NCGC	hspEAsia	complete	4		incomplete	L2R2
VN0244	Vietnam	NCGC	hspEAsia	complete	3	incomplete		
VN0246	Vietnam	NCGC	hspEAsia	complete	4b		complete	L1C1R1
VN0264	Vietnam	NCGC	hspEAsia	complete	3 and 4	incomplete	incomplete	L2
VN0268	Vietnam	NCGC	hspEAsia	complete	4b		complete	L1C1R1
VN0271	Vietnam	NCGC	hspEAsia	complete	3 and 4a	incomplete	incomplete	L2C1R2
VN0272	Vietnam	NCGC	hspEAsia	complete	4		incomplete	L1
VN0274	Vietnam	NCGC	hspEAsia	complete	3	incomplete		
VN0280	Vietnam	NCGC	hspEAsia	complete	4		incomplete	L2R2
VN0283	Vietnam	NCGC	hspEAsia	complete	3	complete		
VN0324	Vietnam	NCGC	hspEAsia	complete	4		incomplete	L2R2
VN0338	Vietnam	NCGC	hspEAsia	complete	3 and 4	incomplete	incomplete	L2C1R1
VN0348	Vietnam	NCGC	hpEurope	complete	3	complete		
VN0352	Vietnam	NCGC	hspEAsia	complete	4		incomplete	L2R2
VN0355	Vietnam	NCGC	hspEAsia	complete	4b		complete	L1C1R1
VN0361	Vietnam	NCGC	hspEAsia	complete	3	complete		
VN0363	Vietnam	NCGC	hspEAsia	complete	4		incomplete	L2R2
VN0366	Vietnam	NCGC	hspEAsia	complete	3 and 4	incomplete	incomplete	L2
VN0382	Vietnam	NCGC	hspEAsia	complete	4		incomplete	L1
VN0390	Vietnam	NCGC	hspEAsia	complete	4		incomplete	L2R2
VN0401	Vietnam	NCGC	hspEAsia	complete	3 and 4	incomplete	incomplete	L2C1R1
VN0403	Vietnam	NCGC	hspEAsia	complete	4		incomplete	L2R2
VN0405	Vietnam	NCGC	hspEAsia	complete	3 and 4	incomplete	incomplete	L2C1R1
VN0406	Vietnam	NCGC	hspEAsia	complete	3 and 4	incomplete	incomplete	L2
VN0410	Vietnam	NCGC	hspEAsia	complete	4b		complete	L1C1R1
VN0411	Vietnam	NCGC	hspEAsia	complete	4		incomplete	L1
VN0418	Vietnam	NCGC	hspEAsia	complete	3 and 4	incomplete	incomplete	L2R2
VN0420	Vietnam	NCGC	hspEAsia	complete	3 and 4	incomplete	incomplete	L2R2
VN0431	Vietnam	NCGC	hspEAsia	complete				
VN0434	Vietnam	NCGC	hspEAsia	complete	4b		complete	L1C1R1
VN0438	Vietnam	NCGC	hspEAsia	complete	4		incomplete	L1
VN0443	Vietnam	NCGC	hspEAsia	complete	3 and 4	incomplete	incomplete	L2
VN0448	Vietnam	NCGC	hspEAsia	complete	4b		complete	L1C1R1
VN0452	Vietnam	NCGC	hspEAsia	complete	4		incomplete	L2
VN0453	Vietnam	NCGC	hspEAsia	complete	4		incomplete	L2R2
VN0464	Vietnam	NCGC	hspEAsia	incomplete	4b		incomplete	L1C1R1
VN0472	Vietnam	NCGC	hspEAsia	complete	4b		complete	L1C1R1
VN0481	Vietnam	NCGC	hspEAsia	complete	4b		complete	L1C1R1
VN0484	Vietnam	NCGC	hspEAsia	complete	3 and 4	incomplete	incomplete	L2
VN0495	Vietnam	NCGC	hspEAsia	complete	4		incomplete	L1
VN0511	Vietnam	DU	hspEAsia	complete	3	complete		
VN0559	Vietnam	DU	hspEAsia	complete	3	complete		
VN0754	Vietnam	DU	hspEAsia	complete	4b		complete	L1C1R1
VN0759	Vietnam	DU	hspEAsia	complete	4b		incomplete	L1C1
VN1154	Vietnam	CG	hspEAsia	complete	3	incomplete	incomplete	
VN1155	Vietnam	CG	hspEAsia	complete	3	incomplete		
VN1156	Vietnam	CG	hspEAsia	complete	4		incomplete	L2R2
VN1158	Vietnam	CG	hspEAsia	complete	3 and 4b	incomplete	complete	L1C1R1
VN1161	Vietnam	CG	hspEAsia		3 and 4	incomplete	incomplete	L2C1R1
VN1163	Vietnam	DU	hspEAsia	complete	3 and 4	complete	incomplete	L2R2
VN1165	Vietnam	CG	hspEAsia		4b		complete	L1C1R1
VN1167	Vietnam	CG	hspEAsia	complete	3 and 4	incomplete	incomplete	L2
VN1169	Vietnam	CG	hspEAsia	complete	3 and 4	incomplete	incomplete	L2C1
VN1171	Vietnam	CG	hspEAsia	complete	3	incomplete		
VN1172	Vietnam	DU	hpEurope		3 and 4	complete	incomplete	L1
VN1175	Vietnam	CG	hspEAsia	incomplete	3 and 4a	incomplete	incomplete	L2C1R2
VN1176	Vietnam	DU	hspEAsia	complete				
VN1178	Vietnam	DU	hspEAsia	complete	4		incomplete	L1
VN1180	Vietnam	DU	hspEAsia	complete	3	complete		
VN1183	Vietnam	CG	hspEAsia	complete	4b		complete	L1C1R1
VN1184	Vietnam	CG	hpEurope	complete	3 and 4c	complete	complete	L2C2R2
VN1186	Vietnam	DU	hspEAsia	complete				
VN1192	Vietnam	CG	hspEAsia	complete	4b		complete	L1C1R1
VN1193	Vietnam	CG	hspEAsia	complete	4		incomplete	L2R2
VN1195	Vietnam	DU	hspEAsia	complete	4		incomplete	L2R2
VN1196	Vietnam	CG	hspEAsia	complete	4b		complete	L1C1R1
VN1197	Vietnam	CG	hspEAsia	complete	4		incomplete	L2R2
VN1199	Vietnam	CG	hspEAsia					
VN1200	Vietnam	CG	hpEurope		3 and 4	complete	incomplete	L1
VN1202	Vietnam	CG	hspEAsia	complete				
VN1203	Vietnam	CG	hspEAsia	complete				
VN1204	Vietnam	CG	hpEurope	complete	3 and 4b	incomplete	incomplete	L1C1R1
VN1205	Vietnam	DU	hspEAsia	complete	4		incomplete	L1
VN1210	Vietnam	CG	hspEAsia	complete	4c		complete	L2C2R2
VN1212	Vietnam	CG	hspEAsia	complete	3 and 4b	incomplete	complete	L1C1R1
VN1213	Vietnam	CG	hspEAsia	complete	4		incomplete	L1
VN1215	Vietnam	DU	hspEAsia	complete	3 and 4	incomplete	incomplete	L2
VN1217	Vietnam	DU	hspEAsia	complete	4		incomplete	L2
VN1218	Vietnam	CG	hspEAsia	complete				
VN1219	Vietnam	CG	hspEAsia	complete	3	incomplete		
VN1221	Vietnam	CG	hspEAsia	complete	4b		complete	L1C1R1
VN1222	Vietnam	DU	hspEAsia	complete	4b		complete	L1C1R1
VN1224	Vietnam	CG	hspEAsia	complete	3 and 4	complete	incomplete	L1
VN1225	Vietnam	CG	hspEAsia	complete	3 and 4a	incomplete	incomplete	L2C1R2
VN1226	Vietnam	CG	hspEAsia	complete				
VN1227	Vietnam	CG	hspEAsia	complete				
VN1229	Vietnam	CG	hpEurope		3 and 4	complete	incomplete	L1
VN1230	Vietnam	DU	hspEAsia	complete	4		incomplete	L2
VN1233	Vietnam	DU	hspEAsia	complete	4		incomplete	L2R2
VN1235	Vietnam	DU	hspEAsia	complete	3 and 4	incomplete	incomplete	L2
VN1236	Vietnam	CG	hspEAsia	complete	4		incomplete	L2C1
VN1237	Vietnam	DU	hspEAsia	complete	4		incomplete	L2
VN1239	Vietnam	CG	hspEAsia	complete	4		incomplete	L2
VN1240	Vietnam	DU	hspEAsia	complete				
VN1241	Vietnam	CG	hspEAsia	complete	3 and 4	complete		L2
VN1244	Vietnam	DU	hspEAsia	complete	3 and 4a	incomplete	incomplete	L2C1R2
VN1246	Vietnam	DU	hspEAsia	complete	4b		complete	L1C1R1
VN1247	Vietnam	CG	hspEAsia	complete	4		incomplete	L2R2
VN1248	Vietnam	DU	hspEAsia	complete				
VN1249	Vietnam	CG	hspEAsia	complete	3 and 4a	incomplete	incomplete	L2C1R2
VN1250	Vietnam	CG	hspEAsia	complete	4		incomplete	L2R2
VN1251	Vietnam	CG	hspEAsia	complete	4b		complete	L1C1R1
VN1258	Vietnam	DU	hspEAsia	complete	4		incomplete	L2R2
VN1260	Vietnam	DU	hspEAsia	complete	3	complete		
VN1261	Vietnam	DU	hspEAsia	complete	3 and 4	complete	incomplete	L2R2
VN1262	Vietnam	DU	hspEAsia	complete	3 and 4c	complete	complete	L2C2R2
VN1263	Vietnam	DU	hspEAsia	complete	3 and 4a	incomplete	complete	L2C1R2
VN1264	Vietnam	DU	hspEAsia	complete	3 and 4	complete	incomplete	L2
VN1266	Vietnam	DU	hspEAsia	complete	3	complete		
VN1269	Vietnam	DU	hspEAsia	complete	4		incomplete	L1C1
VN1270	Vietnam	DU	hspEAsia	complete	3 and 4a	incomplete	incomplete	L2C1R2
VN1272	Vietnam	DU	hspEAsia	complete	3	incomplete		
VN1274	Vietnam	DU	hspEAsia	complete	4		incomplete	L2
VN1276	Vietnam	DU	hspEAsia	complete	3 and 4	incomplete	incomplete	L2R2
VN1277	Vietnam	DU	hspEAsia	complete	3	incomplete		
VN1279	Vietnam	DU	hspEAsia	complete	3	complete		
VN1280	Vietnam	DU	hspEAsia	complete	4		incomplete	L2R2
VN1281	Vietnam	DU	hspEAsia	complete				
VN1283	Vietnam	DU	hspEAsia	complete	3	complete		
VN1284	Vietnam	DU	hspEAsia	complete	4		incomplete	L2R2
VN1286	Vietnam	DU	hspEAsia	complete	3 and 4	incomplete	incomplete	L2R2
VN1288	Vietnam	DU	hspEAsia	complete	4b		complete	L1C1R1
VN1289	Vietnam	DU	hspEAsia	complete				
VN1290	Vietnam	DU	hspEAsia	complete	3 and 4	complete	incomplete	L1
VN1291	Vietnam	DU	hspEAsia	complete	3 and 4c	complete	complete	L2C2R2
**Published genome**		**Accession number**						
MPH01	America	WAEJ00000000	hpWAfrica	complete	3 and 4	incomplete	incomplete	L2C1R1
MPH02	America	WAEI00000000	hpWAfrica	complete	4		incomplete	L2C1
MPH03	America	WAEH00000000	hspEAsia	complete	3 and 4	complete	incomplete	L2
MPH04	America	WAEG00000000	hspEAsia	complete	3 and 4b	complete	complete	L1C1R1
MPH05	America	WAEF00000000	hpWAfrica	complete	3 and 4b	complete	complete	L1C1R1
MPH06	America	WAEE00000000	hpWAfrica	complete	4		incomplete	L2C1R1
MPH08	America	WAED00000000	hpEurope	complete				
MPH09C	America	WAEC00000000	hpWAfrica	complete				
MPH09L	America	WAEB00000000	hpEurope		4		incomplete	L2C1R1
MPH10	America	WAEA00000000	hspEAsia	complete	4b		complete	L1C1R1
MPH11	America	WADZ00000000	hpEurope	complete	3 and 4b	incomplete	complete	L1C1R1
MPH12	America	WADY00000000	hpWAfrica	complete	3 and 4	incomplete	incomplete	L2C1R1
MPH13	America	WADX00000000	hpWAfrica	complete	3 and 4	incomplete	incomplete	L2C1R1
MPH15	America	WADW00000000	hpWAfrica	complete	3 and 4	incomplete	incomplete	L2C1R1
MPH16	America	WADV00000000	hpEurope	complete				
MPH17	America	WADU00000000	hpWAfrica	complete	3 and 4	incomplete	incomplete	L2C1R1
MPH18	America	WADT00000000	hpEurope	complete	4		incomplete	L2C1R1
MPH19	America	WADS00000000	hpEurope		4c		complete	L2C2R2
MPH20	America	WADR00000000	hpEurope	complete				
MPH22	America	WADQ00000000	hpWAfrica	incomplete	3 and 4	complete	incomplete	L2C1R1
MPH23	America	WADP00000000	hpWAfrica	complete	3	complete		
MPH28	America	WADO00000000	hpWAfrica	incomplete	4		incomplete	L2C1R1
MPH29	America	WADN00000000	hpWAfrica	complete	4b		incomplete	L1C1R1
MPH30	America	WADM00000000	hpEurope	complete	4		incomplete	L2C1
MPH31	America	WADL00000000	hpWAfrica	complete	3 and 4	incomplete	incomplete	L2C1R1
MPH32	America	WADK00000000	hpWAfrica	complete	3 and 4	incomplete	incomplete	L2
MPH33	America	WADJ00000000	hpWAfrica	incomplete	3 and 4	incomplete	incomplete	C1R1
MPH34	America	WADI00000000	hpWAfrica	complete	3 and 4b	complete	complete	L1C1R1
MPH35	America	WADH00000000	hpWAfrica	complete	4		incomplete	L2C1
MPH36	America	WADG00000000	hpEurope	complete	3	incomplete		
MPH37	America	WADF00000000	hpEurope		3	incomplete		
MPH38	America	WADE00000000	hpEurope	complete				
MPH39	America	WADD00000000	hpWAfrica	complete	4		complete	L2C1R1
MPH40	America	WADC00000000	hpWAfrica	complete				
MPH41	America	WADB00000000	hpWAfrica	complete	4		complete	L2C1R1
MPH42	America	WADA00000000	hpWAfrica	complete	3 and 4	incomplete	incomplete	L2C1R1
MPH43	America	WACZ00000000	hpWAfrica	complete	3	incomplete		
MPH44	America	WACY00000000	hpWAfrica	complete	3 and 4	complete	incomplete	L2C1R1
MPH47	America	WACX00000000	hpWAfrica	complete	3 and 4	complete	incomplete	L2C1R1
MPH50	America	WACW00000000	hpWAfrica	complete	3 and 4b	incomplete	complete	L1C1R1
MPH51	America	WACV00000000	hpEurope		3 and 4b	complete	complete	L1C1R1
MPH52	America	WACU00000000	hpWAfrica	complete	3 and 4b	incomplete	complete	L1C1R1
MPH54	America	WACT00000000	hpWAfrica		3	incomplete		
KH0001	Cambodia	VEZR00000000	hpEurope	complete				
KH0012	Cambodia	VEZQ00000000	hpEurope	complete	4		incomplete	L2R2
KH0016	Cambodia	VEZP00000000	hspEAsia	complete	3	complete		
KH0022	Cambodia	VEZO00000000	hspEAsia	complete	3 and 4	complete	incomplete	L2
KH0025	Cambodia	VEZN00000000	hpEurope	complete	3 and 4	incomplete	incomplete	L2
KH0031	Cambodia	VEZM00000000	hspEAsia	complete				
KH0032	Cambodia	VEZL00000000	hspEAsia	complete				
KH0033	Cambodia	VEZK00000000	hspEAsia	complete	3 and 4	incomplete	incomplete	L2
KH0040	Cambodia	VEZJ00000000	hpEurope	complete				
KH0042	Cambodia	VEZI00000000	hspEAsia	complete	3 and 4	incomplete	incomplete	L2
KH0045	Cambodia	VEZH00000000	hpEurope	complete	3 and 4a	complete	complete	L2C1R2
KH0054	Cambodia	VEZG00000000	hpEurope	complete	3	complete		
KH0065	Cambodia	VEZF00000000	hpEurope	complete	3 and 4	complete	incomplete	L2C1
KH0066	Cambodia	VEZE00000000	hspEAsia	complete	4		incomplete	L2R2
KH0070	Cambodia	VEZD00000000	hpEurope	complete	3 and 4	complete	incomplete	L2
KH0071	Cambodia	VEZC00000000	hpEurope	complete	3	complete		
KH0072	Cambodia	VEZB00000000	hpEurope		3 and 4a	complete	complete	L2C1R2
KH0075	Cambodia	VEZA00000000	hspEAsia	complete	3 and 4b	incomplete	complete	L1C1R1
KH0086	Cambodia	VEYZ00000000	hpEurope	complete	4		incomplete	L2R2
KH0092	Cambodia	VEYY00000000	hpEurope	complete	3	complete		
KH0095	Cambodia	VEYX00000000	hspEAsia	complete	3 and 4b	complete	incomplete	L1C1R1
KH0097	Cambodia	VEYW00000000	hpEurope	complete				
KH0106	Cambodia	VEYV00000000	hpEurope		3 and 4b	incomplete	complete	L1C1R1
KH0110	Cambodia	VEYU00000000	hspEAsia	complete	4b		complete	L1C1R1
KH0113	Cambodia	VEYT00000000	hspEAsia	complete	3 and 4	incomplete	incomplete	C1R1
KH0119	Cambodia	VEYS00000000	hpEurope	complete	3 and 4	incomplete	incomplete	L2
KH0120	Cambodia	VEYR00000000	hspEAsia	complete	3 and 4	incomplete	incomplete	L2
KH0122	Cambodia	VEYQ00000000	hpEurope	complete				
KH0125	Cambodia	VEYP00000000	hpEurope	complete				
KH0126	Cambodia	VEYO00000000	hpEurope	complete	3 and 4b	incomplete	complete	L1C1R1
KH0127	Cambodia	VEYN00000000	hpEurope	complete	4		incomplete	L2R2
KH0129	Cambodia	VEYM00000000	hpEurope	complete				
KH0141	Cambodia	VEYL00000000	hpEurope	complete				
KH0143	Cambodia	VEYK00000000	hpEurope		4		incomplete	C1R1
KH0148	Cambodia	VEYJ00000000	hpEurope	complete	4		incomplete	L2R2
KH0149	Cambodia	VEYI00000000	hspEAsia	complete	3 and 4	incomplete	incomplete	L2
KH0153	Cambodia	VEYH00000000	hspEAsia	complete	4a		complete	L2C1R2
KH0156	Cambodia	VEYG00000000	hspEAsia	complete	3	incomplete		
KH0158	Cambodia	VEYF00000000	hspEAsia	complete	3 and 4	incomplete	incomplete	L2C1R1
KH0159	Cambodia	VEYE00000000	hpEurope	complete	4		incomplete	L2C1
KH0161	Cambodia	VEYD00000000	hspEAsia	complete	3 and 4	incomplete	incomplete	L1L2C1C2R1
KH0165	Cambodia	VEYC00000000	hpEurope	complete	3	incomplete		
KH0167	Cambodia	VEYB00000000	hspEAsia	complete				
KH0172	Cambodia	VEYA00000000	hpEurope	complete	3	incomplete		
KH0173	Cambodia	VEXZ00000000	hpEurope		4		incomplete	L2
KH0175	Cambodia	VEXY00000000	hspEAsia	complete	4b		complete	L1C1R1
KH0176	Cambodia	VEXX00000000	hpEurope	complete	3	complete		
KH0178	Cambodia	VEXW00000000	hpEurope	complete	3 and 4	incomplete	incomplete	L2
KH0182	Cambodia	VEXV00000000	hspEAsia	complete				
KH0191	Cambodia	VEXU00000000	hspEAsia	complete	4b		complete	L1C1R1
KH0198	Cambodia	VEXT00000000	hpEurope					
KH0207	Cambodia	VEXS00000000	hpEurope	complete	4b		complete	L1C1R1
KH0210	Cambodia	VEXR00000000	hpEurope	complete				

tfs3: virB2	virB3	virB4	virB7	virB8	virB9	virB10	virB11	virD4	virD2	virB6	xerT	ctkA (jhp0940)
[illegible]	[illegible]	[illegible]	[illegible]	[illegible]	[illegible]	[illegible]	[illegible]	[illegible]	[illegible]	[illegible]	[illegible]	[illegible]

tfs4[b] L1: xerT	L1: virB6	L2: xerT	L2: virB6	C1: virD2	C1: virD4	C1: virB11	C1: virB10	C1: virB9	C2: virD2	C2: virD4	C2: virB11	C2: virB10	C2: virB9	R1: virB8	R1: virB7	R1: virB4	R1: virB3	R1: virB2	R2: virB8	R2: virB7	R2: virB4	R2: virB3	R2: virB2
[illegible]	[illegible]	[illegible]	[illegible]	[illegible]	[illegible]	[illegible]	[illegible]	[illegible]	[illegible]	[illegible]	[illegible]	[illegible]	[illegible]	[illegible]	[illegible]	[illegible]	[illegible]	[illegible]	[illegible]	[illegible]	[illegible]	[illegible]	[illegible]

cag pathogenicity island (cagPAI): cagC (virB2)	cagE (virB4)	virB5	cagW (virB6)	cagT (virB7)	cagV (virB8)	cagX (virB9)	cagY (virB10)	cagα (virB11)	cagβ (virD4)
[illegible]	[illegible]	[illegible]	[illegible]	[illegible]	[illegible]	[illegible]	[illegible]	[illegible]	[illegible]

comB: comB2	comB3	comB4	comB7	comB8	comB9	comB10
[illegible]	[illegible]	[illegible]	[illegible]	[illegible]	[illegible]	[illegible]

[a]*tfs3* ICE genes were identified by sequence comparison with *tfs3* ICE retrieved from strain Gambia94/24 and strain India7 and represented by gene coverage and identity percentage
[b]*tfs4* ICE genes were identified by sequence comparison with *tfs4a* (L2C1R2), *tfs4b* (L1C1R1), *tfs4c* (L2C2R2) retrieved from *H. pylori* strain P12, Shi470 and R036d represented by gene coverage and identity percentage
[c]*tfs3* ICE complete were defined when there were presence of *virB2, virB3, virB4, virB6, virB8, virB9, virB10, virB11, virD2* and *virD4*
[d] *tfs4* ICE complete were defined when there were presence of *virB2, virB3, virB4, virB6, virB8, virB9, virB10, virB11, virD2* and *virD4*
[e]Gastroduodenal diseases patients were divided into 3 groups: NCGC (non-cardia gastric cancer), DU (duodenal ulcer), and CG (chronic gastritis)
[f]Geographical population was assigned into each strains based on MLST of 7 house-keeping genes of *H. pylori*
[g]Classification of *tfs* ICE was based on nucleotide identity >80% compared to *tfs* ICE of reference strains

Table S7: Distribution of *tfs3/4* ICE on geographical populations in Vietnam

		hpEurope (%), n=6	hspEAsia (%), n=130
tfs3		6 (100)	56 (43)
tfs4 module	L1	4 (66)	37 (28)
	L2	1 (16)	63 (48)
	C1	1 (16)	39 (30)
	C2	1 (16)	3 (23)
	R1	1 (16)	26 (20)
	R2	1 (16)	37 (28)
tfs4		5 (83)	100 (76)
tfs ICE positive		6 (100)	117 (90.0)
